# Extracellular vesicles shuttle protective messages against heat stress in bovine granulosa cells

**DOI:** 10.1038/s41598-020-72706-z

**Published:** 2020-09-25

**Authors:** Samuel Gebremedhn, Ahmed Gad, Hoda Samir Aglan, Jozef Laurincik, Radek Prochazka, Dessie Salilew-Wondim, Michael Hoelker, Karl Schellander, Dawit Tesfaye

**Affiliations:** 1 Animal Reproduction and Biotechnology Laboratory, Department of Biomedical Sciences, Colorado State University, 1351 Rampart Rd, Fort Collins, CO 80525 USA; 2 Animal Breeding and Husbandry Group, Institute of Animal Science, University of Bonn, Bonn, Germany; 3 Laboratory of Developmental Biology, Institute of Animal Physiology and Genetics, Czech Academy of Sciences, Liběchov, Czech Republic; 4 Department of Animal Production, Faculty of Agriculture, Cairo University, Giza, Egypt; 5 Constantine the Philosopher University in Nitra, Nitra, Slovakia; 6 Department of Animal, Rangeland and Wildlife Sciences, Mekelle University, Mekelle, Ethiopia

**Keywords:** Biotechnology, Cell biology, Developmental biology, Molecular biology

## Abstract

Elevated summer temperature is reported to be the leading cause of stress in dairy and beef cows, which negatively affects various reproductive functions. Follicular cells respond to heat stress (HS) by activating the expression of heat shock family proteins (HSPs) and other antioxidants. HS is reported to negatively affect the bi-directional communication between the follicular cells and the oocyte, which is partly mediated by follicular fluid extracellular vesicles (EVs) released from surrounding cells. As carriers of bioactive molecules (DNA, RNA, protein, and lipids), the involvement of EVs in mediating the stress response in follicular cells is not fully understood. Here we used an in vitro model to decipher the cellular and EV-coupled miRNAs of bovine granulosa cells in response to HS. Moreover, the protective role of stress-related EVs against subsequent HS was assessed. For this, bovine granulosa cells from smaller follicles were cultured in vitro and after sub-confluency, cells were either kept at 37 °C or subjected to HS (42 °C). Results showed that granulosa cells exposed to HS increased the accumulation of ROS, total oxidized protein, apoptosis, and the expression of HSPs and antioxidants, while the viability of cells was reduced. Moreover, 14 and 6 miRNAs were differentially expressed in heat-stressed granulosa cells and the corresponding EVs, respectively. Supplementation of stress-related EVs in cultured granulosa cells has induced adaptive response to subsequent HS. However, this potential was not pronounced when the cells were kept under 37 °C. Taking together, EVs generated from granulosa cells exposed to HS has the potential to shuttle bioactive molecules to recipient cells and make them robust to subsequent HS.

## Introduction

Over the past decades, global climate change caused an increase in the atmospheric CO_2_ release and average daily temperature, which resulted in more frequent heat waves during the summer seasons. The increase in the ambient temperature during the summertime is the leading cause of stress in dairy production, which causes a decline in reproductive and production performances of dairy cows^1,2^. Heat stress (HS) negatively affects the fertility of dairy cows by disrupting the follicular development, ovarian functions, steroidogenesis, fertilization, early embryonic development, and the maternal recognition of the pregnancy^3–6^. Besides, exposure of cows to summer HS has a long-lasting impact and could fully recover from the induced damages after at least 2–3 estrous cycles^7^. Bovine oocytes exposed to HS during the maturation period showed impairment in the rearrangement of the microtubules and microfilaments, accompanied by damaged spindle apparatus, and significant increment in the proportion of oocytes arrested at metaphase, leading to lower fertilization rate^8,9^.

At the cellular level, mammalian cells respond to HS in various ways, including the activation of the heat shock proteins (HSPs)^10^, unfolded protein response (UPR), and oxidative stress responses^11^. Exposure of cells to HS induces the accumulation of intracellular reactive oxygen species (ROS)^12^, which in turn causes apoptosis^13^. The HSPs work as chaperons and interact with other proteins to influence the folding, trafficking, and degradation^14^. The duration of exposure to HS could determine the specific detrimental effect it poses to the cells. Chronic heats stress (long-term exposure to heat) leads to the induction of the HSPs and gradual adaptation of cells to high temperature^15^. On the other hand, acute HS (short term, sub-lethal exposure of cells to heat) causes significant damage and leads to the rapid synthesis and upregulation of the HSPs to facilitate the folding and transportation of degraded proteins^14^. The HSPs are categorized into several groups according to their molecular size and functions. Among the HSP family, the HSP70 is the major stress protein elevated in response to HS in bovine^16^ and mouse^17^ granulosa cells. Moreover, the HSP70 engages the HSP90 for its folding and degradation^18^. Elevated expression of HSP70 reduces the aromatase protein in antral follicles, which is associated with a decline in the fertility of animals exposed to HS^19^. Similarly, the GRP78 and GRP94, which are reported to be abundantly expressed in granulosa cells of antral follicles belong to the HSP family and are localized in the Endoplasmic Reticulum (ER) lumen^20^.

Bi-directional communication between the follicular cells and oocyte is required during the process of folliculogenesis^21^. There are several mechanisms of cell-to-cell communications including via hormones and cytokines, surface contact molecules^22^, and exchange of metabolites of lower molecular weight via gap junction^23^. Another mechanism of cell-to-cell communication is via membrane vesicles, which contain surface molecules and cargo of bioactive molecules. Extracellular Vesicles (EVs) are naturally occurring submicron-sized, lipid bilayer membrane-enclosed complexes released by cells into the surrounding extracellular space. According to the International Society of Extracellular Vesicles (ISEV), the EVs can be used as a generic terminology to describe heterogeneous mixtures of vesicles due to the technical difficulties to isolate and characterize a distinct subtype of vesicles^24^. EVs serve the cells either as tools to shuttle excess or unnecessary cellular constituents to maintain cellular homeostasis or mediators of intracellular communication^25^. The surface of EVs contains ligands, which acts as recognition signals direct to the receptors of target cells. EVs are loaded with bioactive molecules including DNA, RNAs, lipids, metabolites, and proteins of cytosolic and surface proteins of the originating cells^26^, and upon their uptake by recipient cells, they deliver these bioactive molecules and pose phenotypic changes^27^. EVs are present virtually in all reproductive biofluids including follicular fluid^28–30^, oviductal fluid^31^, and spent culture medium of follicular cells^32,33^. Under normal physiological conditions, follicular cells communicate to maintain the homeostasis of the follicular microenvironment, which leads to the release of a developmentally competent oocyte. Likewise, when follicular cells are exposed to environmental and metabolic stressors, intracellular communication is maintained by shuttling stress signals to neighboring cells to adjust the metabolism to the new stressful environment^34^. Oocytes exposed to HS had significantly reduced the cumulus cell expansion, cleavage rate, and percentage of the oocyte that reached the blastocysts after fertilization^35^. In the same study, it was shown that supplementation of both follicular fluid and exosomes isolated from follicular fluid reversed the damage of HS on the oocyte and improved the cleavage and blastocyst rates. Interestingly, supplementation oocytes with only exosomes derived from follicular fluid have a better impact than the supplementation of the whole follicular fluid. This signifies the fact that exosomes play a major role in carrying molecular signals with a potential role in reversing or protecting the damage incurred by HS on the oocytes.

The HSPs are among the stress signals released by cells via EVs upon exposure to HS. The HSP70 is reported to be released from cells exposed to HS and the release of the protein could not be blocked by pharmacological inhibition of the regular secretory pathways^36^. Similarly, the HSP90^37,38^ and GRP78^39^ were also detected in the extracellular space released via EVs. Granulosa cells exposed to oxidative stress release EVs enriched with the NRF2 transcription factor and its downstream antioxidants, CAT and TXN1^32^. Interestingly, in the same study, it has been revealed that EVs primed with oxidative stress could rescue cells under oxidative stress conditions and improve their viability. Similarly, EVs obtained from the follicular fluid of cows with divergent metabolic status were found to be loaded with unique miRNA molecular signatures^30^ associated with altered the epigenome profile of the accompanying oocytes^40^. Such in vivo studies cannot provide a clear picture of the effect of environmental or physiological stress in specific follicular cells. Therefore, the application of an in vitro granulosa cell culture model would enable us to investigate the effect of HS on this specific follicular cell type and their response by releasing EV-coupled molecules into extracellular space. Here we aimed to decipher the molecular contents of EVs released from bovine granulosa cells exposed to HS and evaluate the protective role of these EVs against subsequent HS.

## Results

### HS induced ROS accumulation and reduced the viability of granulosa cells

**Figure 1:**
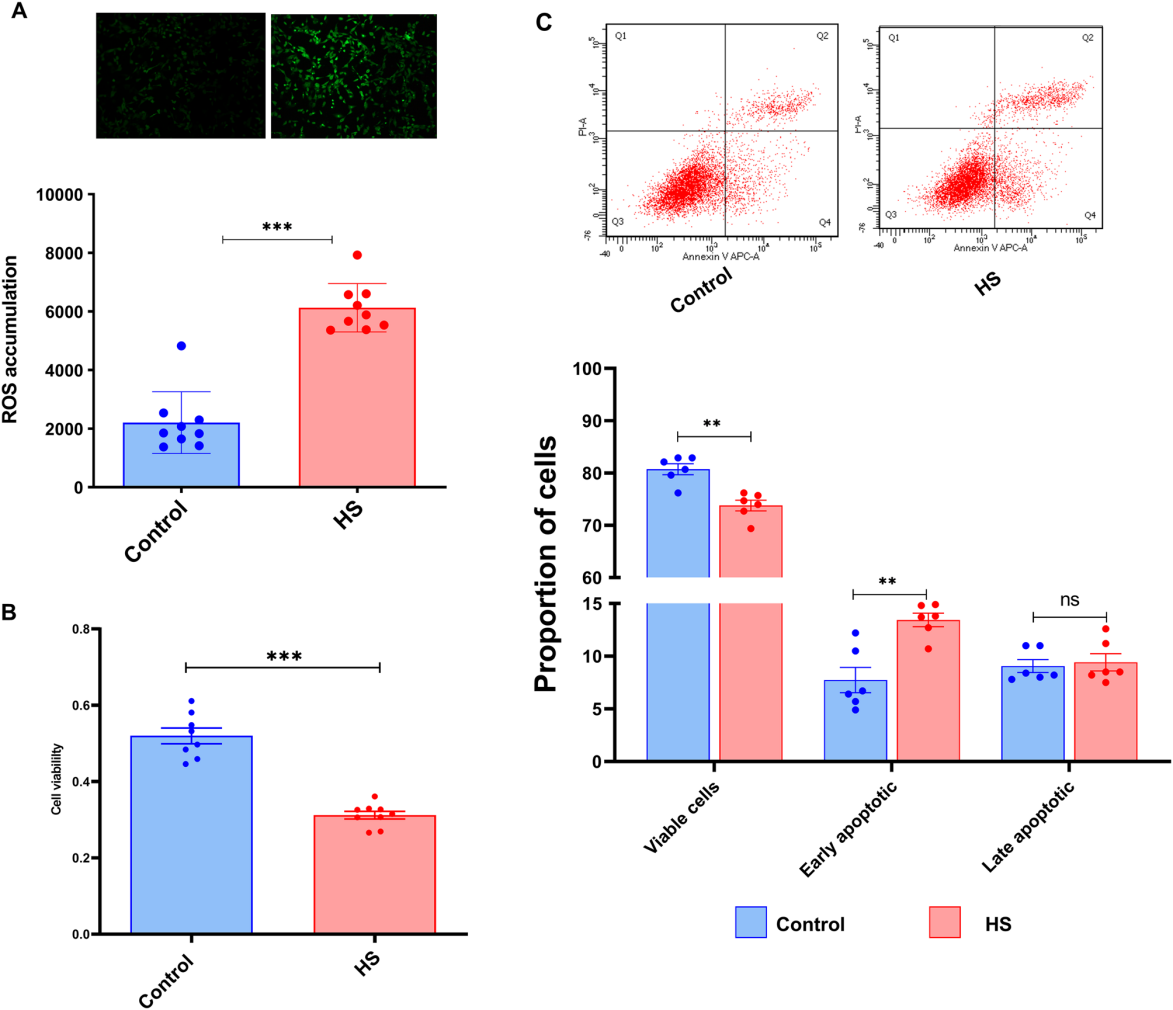
Impact of HS on bovine granulosa cells. The ROS level of bovine granulosa cells exposed to HS was analyzed using the ROS staining technique. Representative pictures are shown on the upper panels and shows a strong green fluorescence signal in granulose cells subjected to HS. The summary histogram showed significant accumulation of ROS in the HS group (**A**). The viability of granulosa cells subjected to HS was significantly reduced (**B**). Annexin V/PI staining showed a significant reduction in the proportion of live cells and the significant increment in the proportion of early apoptotic cells in the HS group. The upper panels show the distribution of cells stained for both Annexin V and PI across the different quadrants (**C**). Data are presented as mean ± SEM and the mean differences were analyzed using the Two-tail student’s *t* test. *p < 0.05; **p < 0.01; ***p < 0.001 and *ns* not significant.

To determine the impact of heat stress, granulosa cells were incubated under HS (42 °C) and normal incubating temperature (37 °C). It was shown that exposure of granulosa cells to HS resulted in a significant increase in the accumulation of intracellular ROS compared to the control counterparts (Fig. 1A). Contrary to this, the cell viability was significantly reduced upon exposure to HS (Fig. 1B). This was further examined using the Annexin V/PI staining to determine the exact apoptosis status of cells subjected to HS. Moreover, exposure of granulosa cells to HS compromised the membrane integrity and significantly decreased the proportion of viable cells and significantly increased the proportion of early apoptotic cells compared to the cells kept under the control conditions (Fig. 1C).

### HS induced the expression of the HSPs and other stress-related transcripts in bovine granulosa cells

**Figure 2:**
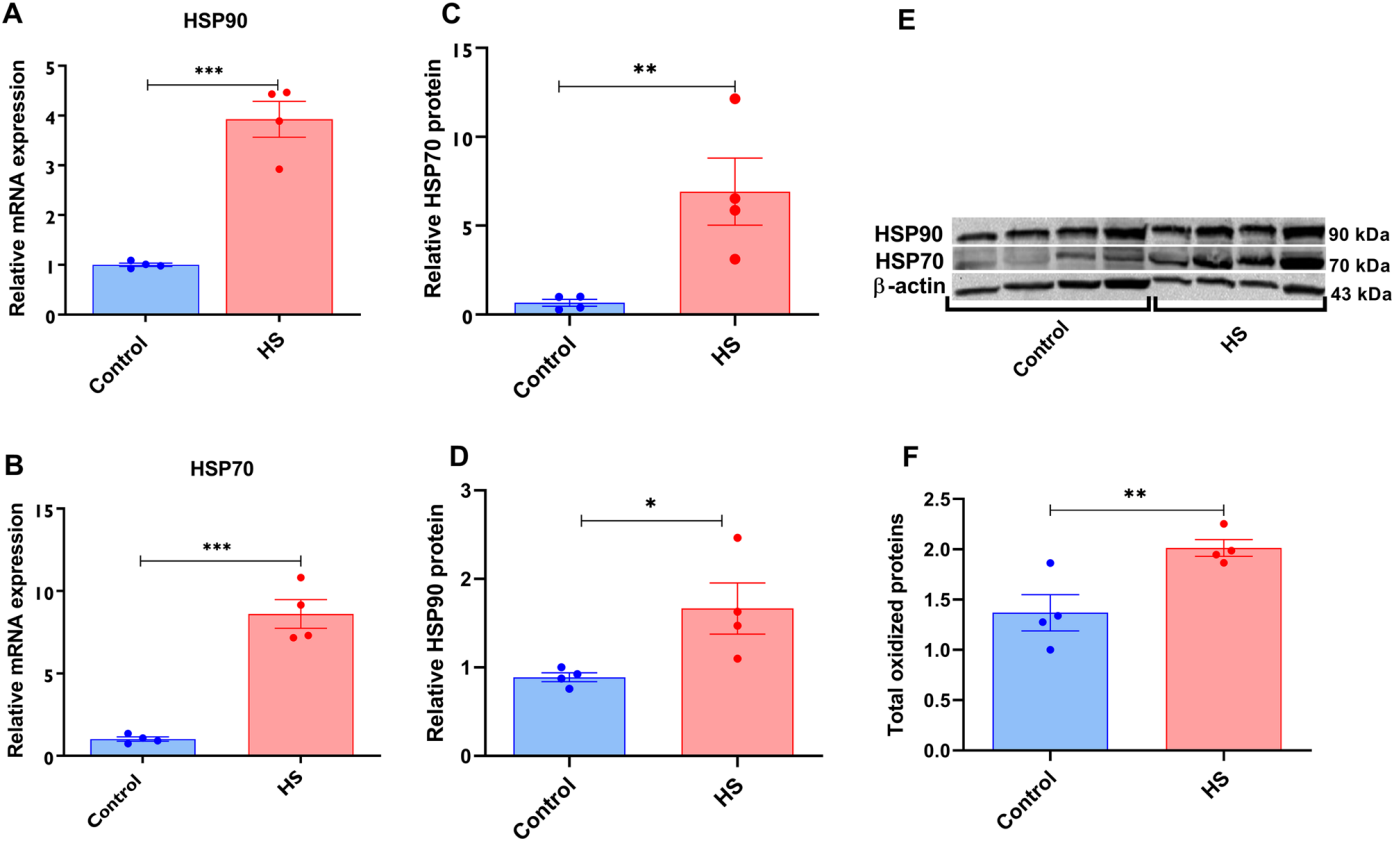
Impact of HS on the expression of HSPs and protein oxidation. The transcript and protein abundance of the HSPs using qRT-PCR and western blot, respectively. Elevated expression of the HSP90 (**A**), HSP70 (**B**) was observed in HS cells. The summary of the western blot analysis showed significant upregulation of the HSP90 (**C**) and HSP70 (**D**). The band strength of the HSP90 and HSP70 in each replicate is also indicated (**E**). The impact of HS on the amount of total oxidized protein was examined and cells exposed to HS had a significantly higher amount of oxidized proteins (**F**). Data are presented as mean ± SEM and the mean differences were analyzed using the Two-tail student’s *t* test. *p < 0.05, **p < 0.01; ***p < 0.001. Full-length blots are presented in Supplementary Figure S5.

**Figure 3:**
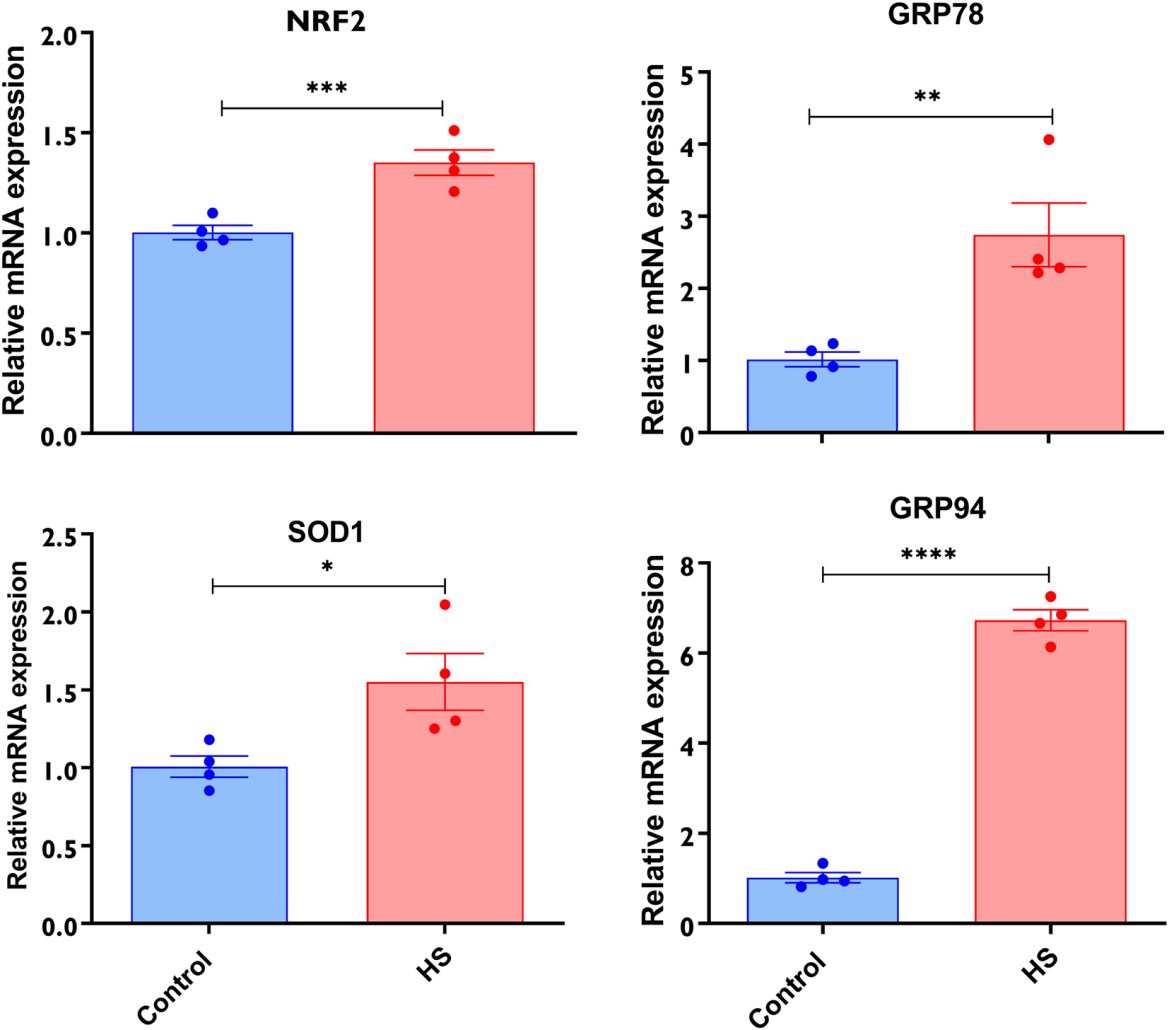
Expression of stress-associated genes in granulosa cells subjected to HS. The impact of HS on the expression of genes associated with oxidative stress (NRF2 and SOD1) and endoplasmic reticulum stress (GRP94 and GRP78) was assessed using qRT-PCR. The expression of the genes was significantly higher in HS granulosa cells. Data are presented as mean ± SEM and the mean differences were analyzed using the Two-tail student’s *t* test. *p < 0.05; **p < 0.01; ***p < 0.001; ****p < 0.0001.

The impact of HS on the expression level of the HSPs mRNAs and proteins and other stress-related transcripts were evaluated. The relative mRNA abundance of HSP90 and HSP70 was significantly upregulated in response to HS compared to the cells maintained at 37 °C (Fig. 2A,B). Besides, the proteins abundances of the HSPs were also elevated in response to HS (Fig. 2C, D, and E). In addition to the elevated HSP transcripts and proteins, it was shown that granulosa cells exposed to HS had a significantly higher abundance of total oxidized proteins (Fig. 2F). To determine the impact of HS on the oxidative and ER stress response of cells, transcripts associated with oxidative stress (NRF2 and SOD1) and ER stress (GRP78 and GRP94) were examined and the expression of all the transcripts was significantly enriched in granulosa cells subjected to HS (Fig. 3).

### HS enhanced the release of EVs in bovine granulosa cells

**Figure 4:**
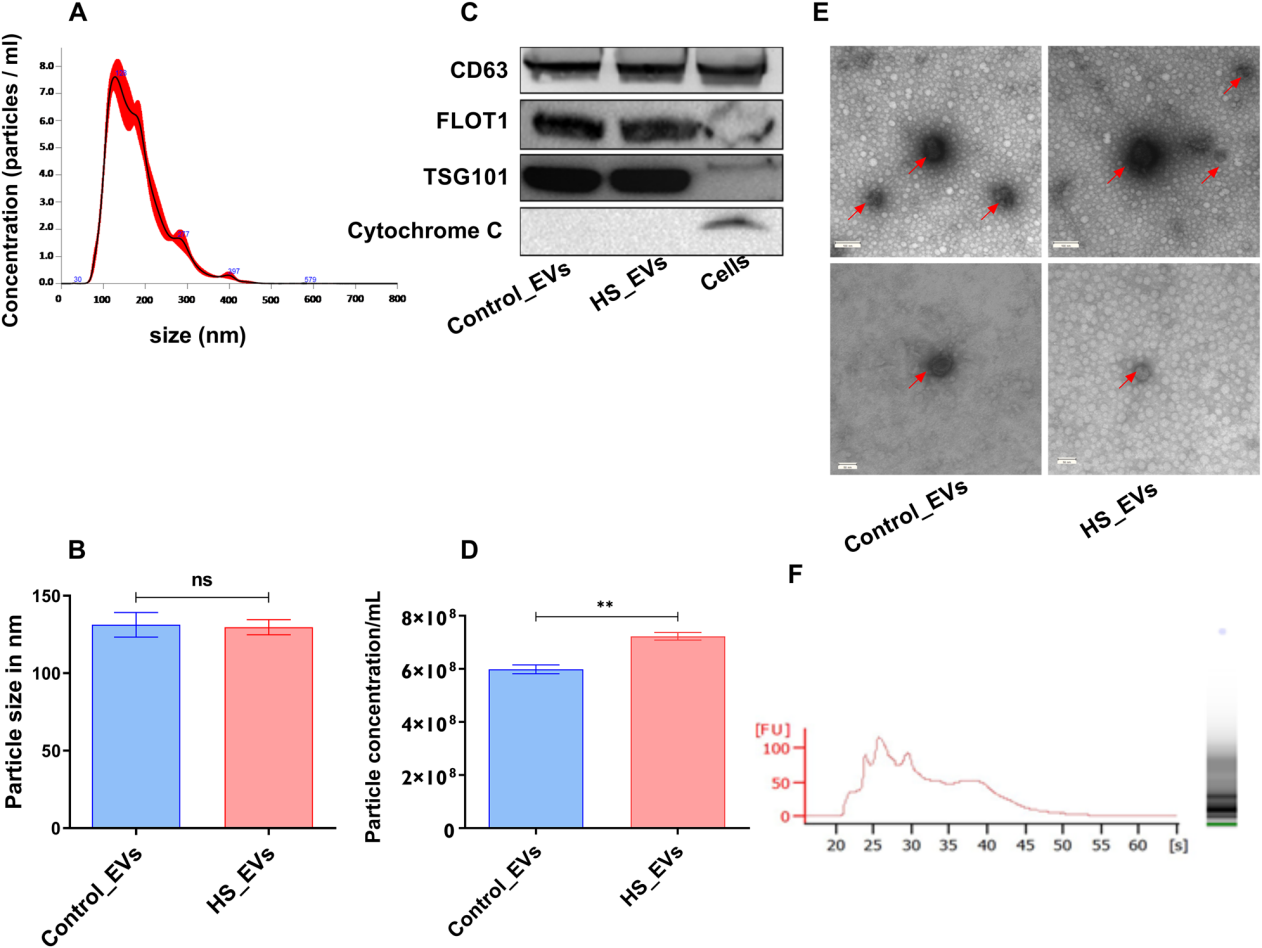
Morphological and molecular characterization of EVs: The size of the EVs isolated from the spent culture media of granulosa cells using NTA analysis and the mode size was between the range of 120–130 nm (**A**) and no significant difference was observed in the size of the EVs released from granulosa cells cultured under both temperatures (**B**). Immunoblotting analysis of EVs marker protein CD63, FLOT1, and TSG101 and cellular contamination indicator CYCS. The CD63, FLOT1, and TSG101 were detected in the EVs and the cellular protein lysates, However, the CYCS was not detected in the EVs, but detected only in the cellular protein sample lane. Full-length blots are presented in Supplementary Figure S6 (**C**). The concentration of EVs released from granulosa cells was quantified using the NTA analysis. Granulosa cells subjected to HS release significantly more EVs than the cells kept at 37 °C (**D**). Transmission electron microscope (TEM) shows a clear morphology of EVs (indicated by red arrows) from both the control and HS group. The upper panels indicate EVs with a scale bar of 100 nm and the lower bars indicate a scale bad of 50 nm (**E**). The RNA size distribution of EVs demonstrated the presence of the peak of small RNA molecules and the absence of the 18 s and 28 s cellular RNA components (**F**). Data are presented as mean ± SEM and the mean differences were analyzed using the Two-tail student’s *t* test. **p < 0.01, *ns* not significant.

**Figure 5:**
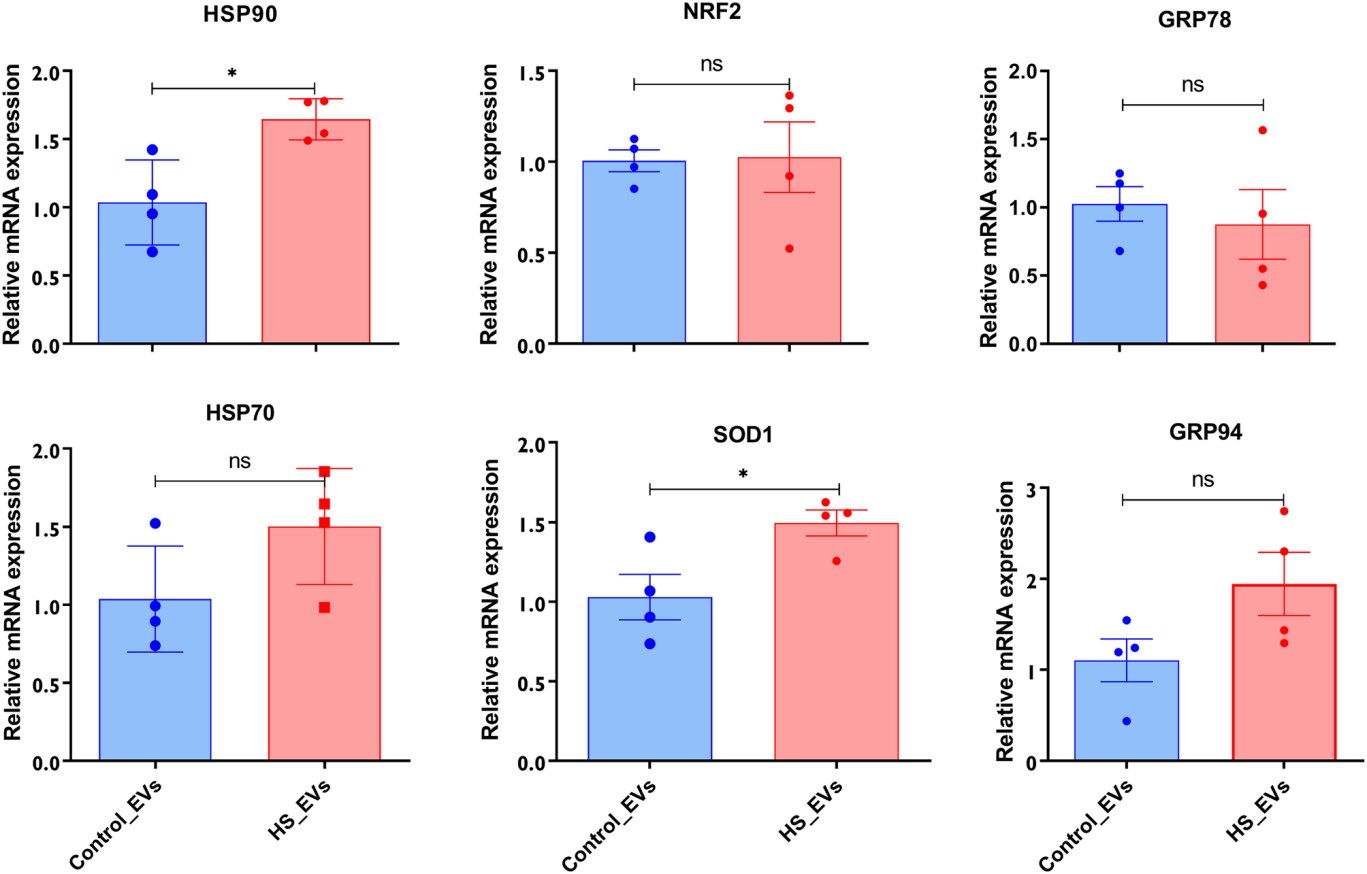
Expression of stress-associated genes in EVs released from granulosa cells subjected to HS. The impact of HS on the expression of stress-associated genes encapsulated in the EVs was assessed using qRT-PCR. The expression of the HSP90 and SOD1 were significantly enriched in HS-EVs. The expression of HSP70 and GRP94 tends to be higher in HS-EVs, but statistically not significant. There was no significant difference in the expression of NRF2 and GRP78 between the control and the HS-EVs. Data are presented as mean ± SEM and the mean differences were analyzed using the Two-tail student’s *t* test. *p < 0.05, *ns* not significant.

The spent culture media of granulosa cells exposed to HS and those kept under 37 °C (Controls) were subjected to EVs isolation. Morphological and molecular characterization of the isolated EVs was performed based on the standards set by the ISEV. Assessment of the size and concentration of the EVs using the Nanoparticle Tracking Analysis (NTA) showed that the EVs released from both HS and control groups were within the range of 120–130 nm of diameter (Fig. 4A) and no measurable differences were observed in the diameter of the EVs posed due to HS (Fig. 4B). Western blotting confirmed that EVs from both HS and control groups were positive for the transmembrane protein CD63, FLOT1, and TSG101, and negative for mitochondrial protein marker CYCS (Fig. 4C), verifying the identity of the EVs and suggesting the absence of contamination of the EVs with cellular debris. There was a significantly higher number of EVs that were released from granulosa cells subjected to HS compared to those kept at 37 °C (Fig. 4D). Similarly, TEM imaging showed the presence of multiple EVs with visible bilipid membranes (Fig. 4E). In addition, the RNA size distribution according to the RNA electropherogram of RNA isolated from EVs showed the absence of 18 s and 28 s, while the picks show the presence of small RNAs in the RNA samples of the EVs (Fig. 4F), signifying the absence of cellular contamination during the EVs isolation procedure. The encapsulation and release of stress-associated transcripts via EVs were assessed using qRT-PCR and it was revealed that EVs released from granulosa cells under HS conditions (HS-EVs) contain significantly higher mRNA levels of HSP90 and SOD1. In addition, the tendency of the expression of HSP70 and GRP94 was higher in HS-EVs compared to the EVs obtained from unstressed control cells (Control-EVs) (Fig. 5). This signifies that EVs are involved in shuttling stress-associated gene transcripts in bovine granulosa cells upon exposure to HS.

### Granulosa cells subjected to HS and the corresponding EVs exhibited divergent miRNAs expression profile

The impact of HS on the expression of cellular and the EV-coupled miRNAs was examined by next-generation sequencing. We set a stringent criterion to determine the detection of miRNAs in which, a miRNA with an average normalized read count of 10 and above in at least two of the biological triplicates was regarded as detected. Accordingly, a total of 274 miRNAs were detected in both HS and the control granulosa cells, of which 269 were commonly expressed, while 5 miRNAs from each group were preferentially detected in each group (Supplementary Fig. S2-A). Similarly, a total of 187 and 194 miRNAs were detected in HS-EVs and control-EVs, respectively. Of these 10 and 17 miRNAs were preferentially released in HS-EVs and control-EVs, respectively. It is worth noting, that majority of the miRNAs were commonly detected in both granulosa cells and the corresponding EVs released to the extracellular space. Principal component analysis (PCA) on the normalized expression values of the miRNAs in both granulosa cells and EVs was performed. It was shown that there was a clear separation between granulosa cells exposed to HS and cells maintained at 37 °C, and the first two principal components explained about 58.5% of the existing variances (Supplementary Fig. S2-B). Similarly, PCA on the miRNAs data of the EVs showed a clear separation between the HS-EVs and control-EVs, and the first two principal components explain 45.3% of the variations (Supplementary Fig. S2-C). The sequence statistics and number of reads passed the QC parameters and mapped to the bovine genome and miRbase database are indicated in supplementary table S2.

### Granulosa cells subjected to HS displayed differential expression of the cellular and EVs-coupled miRNAs

**Figure 6:**
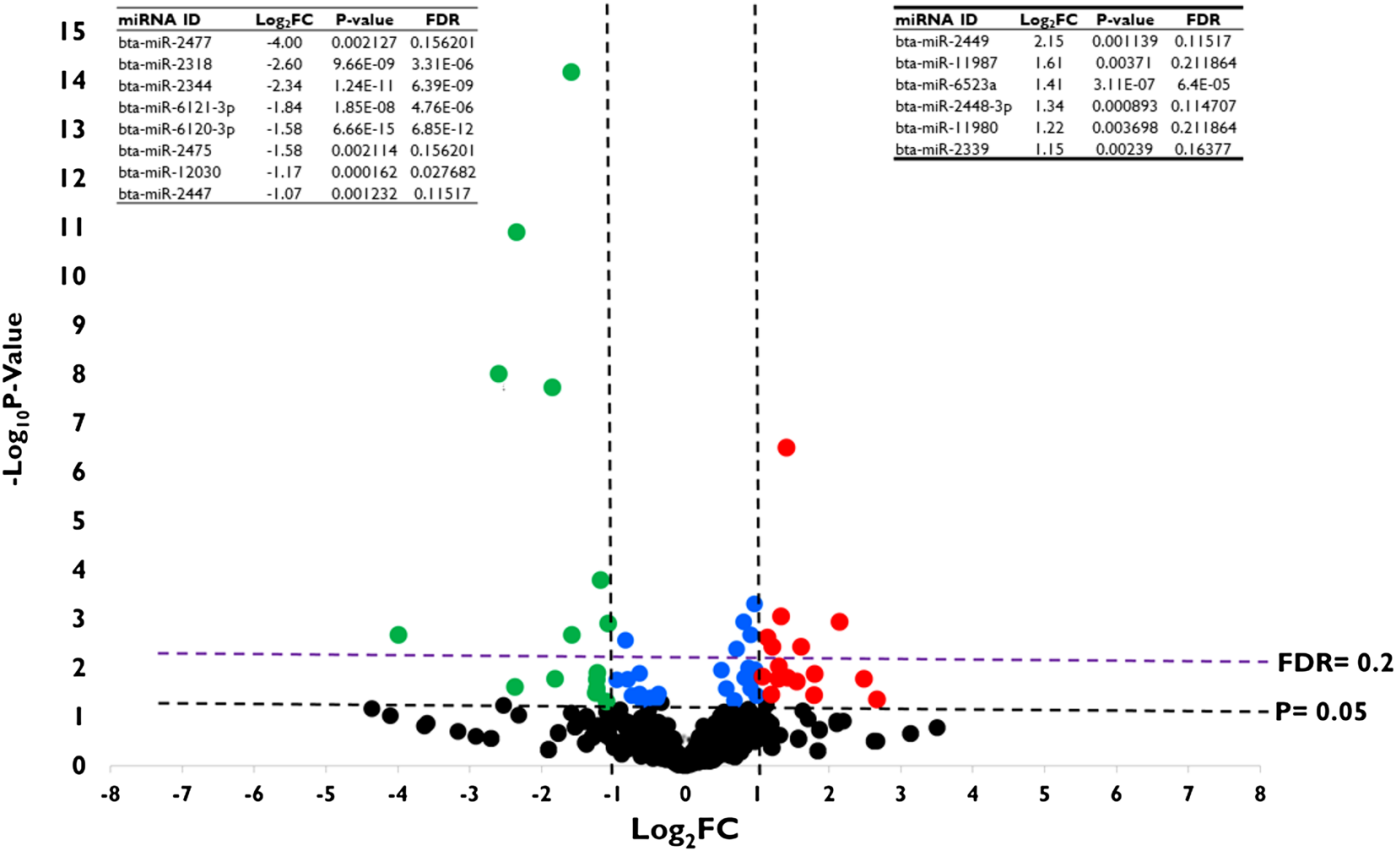
Differential expression of miRNAs in granulosa cells subjected to HS. Volcano plot displaying differentially expressed miRNAs in granulosa cells exposed to HS. Each dot displayed in the plot represents a single miRNA and miRNAs upregulated and downregulated in granulosa cells exposed to HS and are denoted with red and green dots, respectively. The blue dots represent miRNAs, which have lower fold change, but statistically significant. Statistical significance is determined at log_2_FC >|1| and FDR of 0.2.

**Figure 7:**
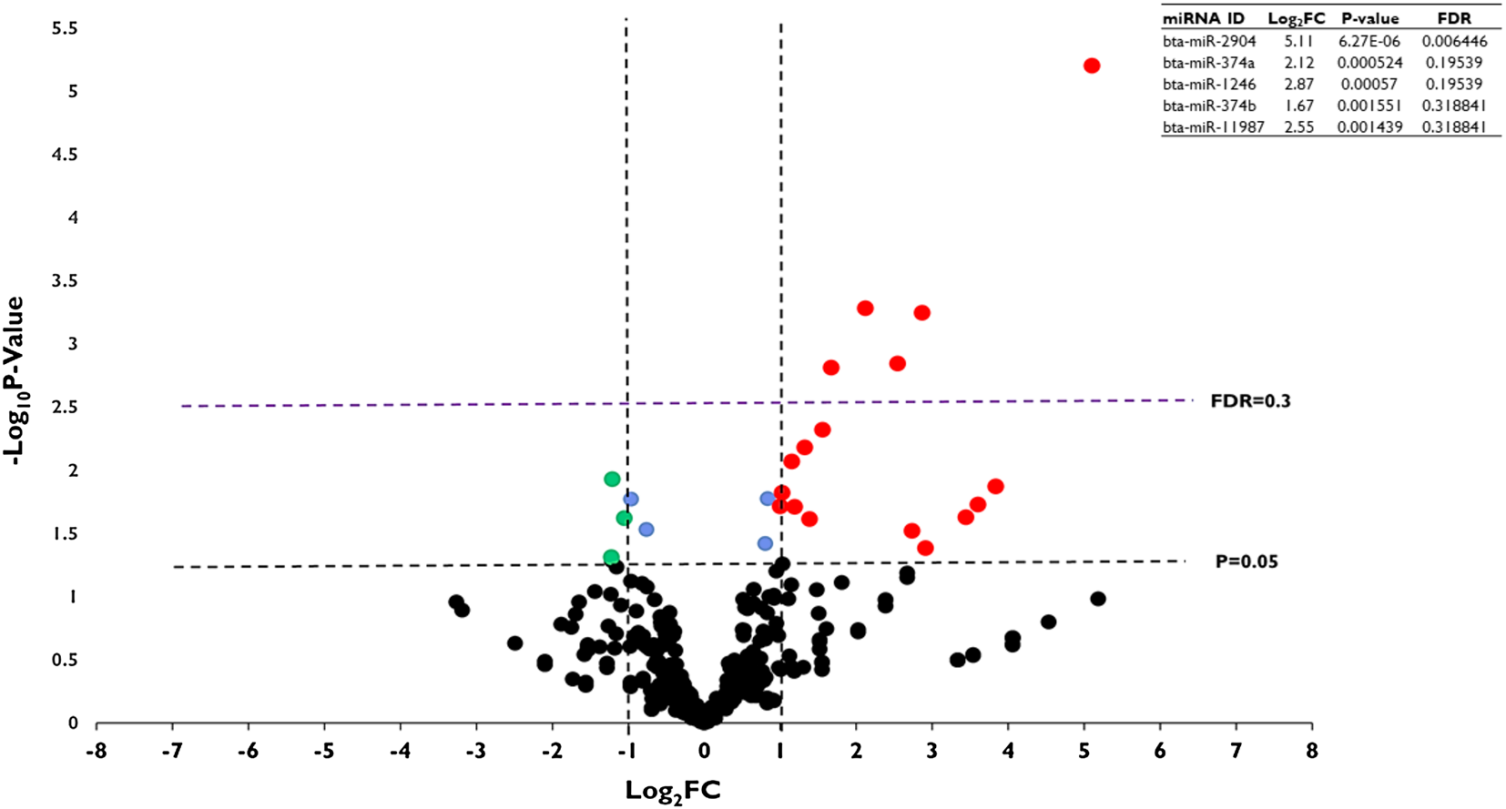
Differential expression of miRNAs in the HS-EVs. Volcano plot displaying differentially expressed miRNAs in HS-EVs. Each dot displayed in the plot represents a single miRNA and miRNAs upregulated and downregulated in HS-EVs are denoted with red and green dots, respectively. The blue dots represent miRNAs, which have lower fold change, but statistically significant. Statistical significance is determined at log_2_FC >|1| and FDR of 0.3.

To determine the impact of HS on the expression of miRNAs, the expression level of miRNAs was assessed in both HS and control groups. It was revealed that most of the miRNAs are equally expressed between both groups. However, 14 miRNAs were found to be differentially expressed (Padjusted value of ≤ 0.2 and Log_2_FC value of ≥|1|) between the two groups, of which 6 miRNAs (bta-miR-2449, bta-miR-11987, bta-miR-6523a, bta-miR-2448-3p, bta-miR-11980, and bta-miR-2339) were significantly upregulated and 8 other miRNAs (bta-miR-2477, bta-miR-2318, bta-miR-2344, bta-miR-6121-3p, bta-miR-6120-3p, bta-miR-2475, bta-miR-12030 and bta-miR-2447) were significantly downregulated in granulosa cells exposed to HS compared to those incubated at 37 °C (Fig. 6). The validation of the expression of miR-2344, bta-miR-6523a, and bta-miR-6120-3p using ddPCR agrees with the sequencing data (Supplementary Fig. S3-A). Similarly, EVs released from HS challenged granulosa cells are enriched with distinct sets of miRNAs. Accordingly, 5 miRNAs (bta-miR-2904, bta-miR-374a, bta-miR-1246, bta-miR-374b and bta-miR-11987) were significantly enriched (Padjusted value of ≤ 0.3 and Log_2_FC value of ≥|1|) in HS-EVs compared to the control-EVs (Fig. 7). The ddPCR validation showed that, among the tested, three differentially expressed miRNAs (bta-miR-2904, bta-miR-374a, bta-miR-1246), only the expression of bta-miR-1246 was in agreement with the sequencing result (Supplementary Fig. S3B). A list of all differentially expressed miRNAs in granulosa cells subjected to HS and the corresponding EVs is indicated in Supplementary Tables S3 and S4.

### In silico target gene prediction, gene ontology, and pathway analysis

**Figure 8:**
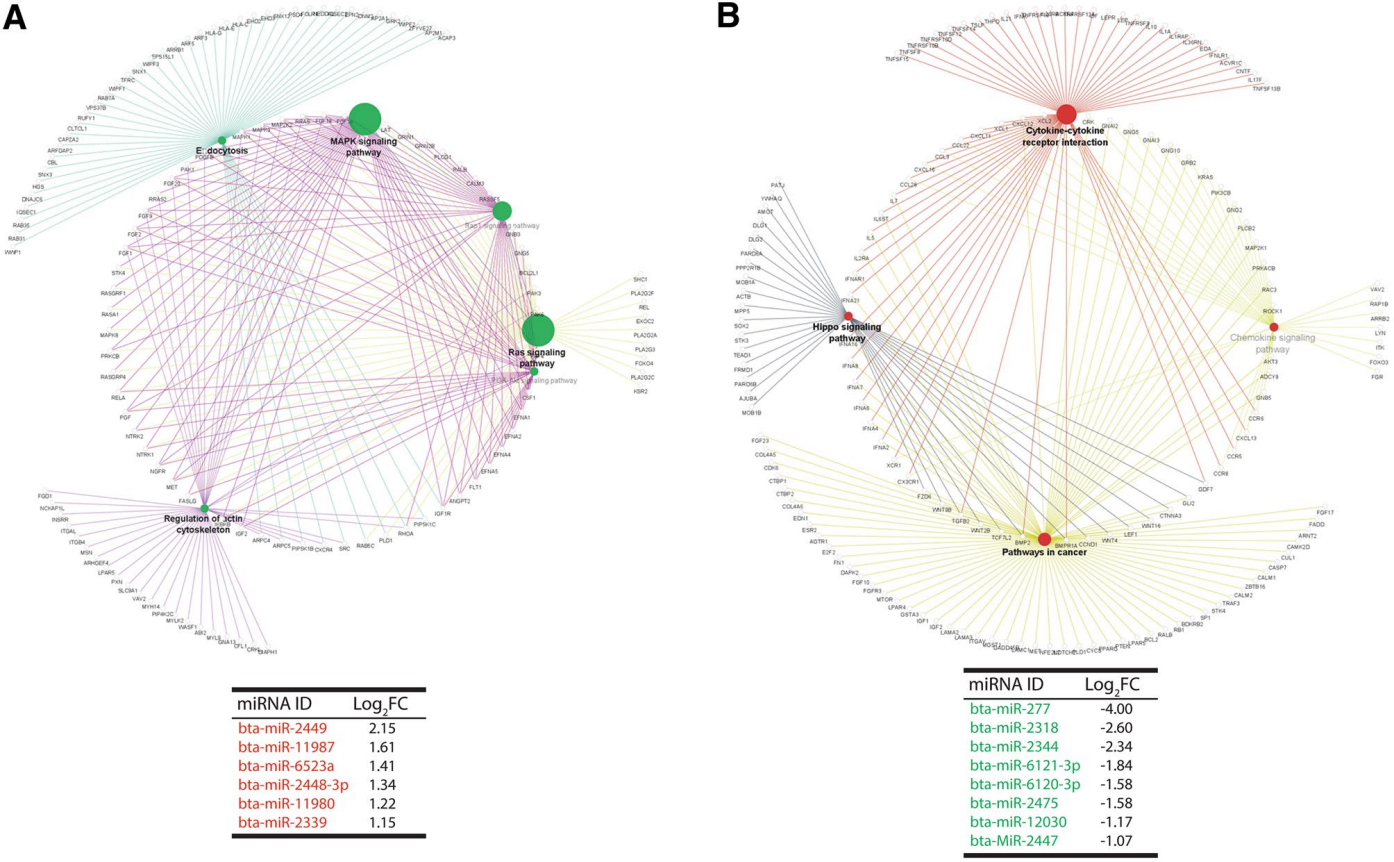
Target gene prediction and pathway analysis. Interactive networks between signaling pathways enriched by the predicted target genes miRNAs significantly upregulated (**A**) and down-regulated (**B**) in granulosa cells exposed to HS.

Genes predicted to be targeted by the differentially expressed miRNAs in HS granulosa cells were subjected to pathway analysis and ontological classifications. A total of 3712 and 2824 genes were predicted to be targeted by the miRNAs up and down-regulated in HS granulosa cells, respectively. Among the predicted genes, 148 and 134 were commonly targeted by at least two of the up and down-regulated miRNAs, respectively. Pathway analysis on the predicted target genes revealed that the MAPK signaling pathway, Ras signaling pathway, Rap1 signaling pathway, endocytosis, regulation of actin cytoskeleton and the PI3K-AKT signaling pathways are the top pathways enriched by the predicted target genes of HS induced miRNAs in granulosa cells (Fig. 8A). The cytokine-cytokine receptor interaction, pathways in cancer, the Hippo signaling pathway, and the chemokine signaling pathway were among the most significant pathways enriched by predicted target genes of miRNAs downregulated in HS granulosa cells (Fig. 8B). Similarly, miRNAs enriched the EVs released from HS bovine granulosa cells are predicted to target a total of 1002 genes, among which 23 genes were commonly predicted to be targeted by at least two of the differentially expressed miRNAs. The pathways analysis revealed that the predicted target genes are involved in metabolism pathways (glutathione metabolism, retinol metabolism, glycerol phospholipid metabolism, cholesterol metabolism, and others), glucagon signaling pathway, AMPK signaling pathway and circadian rhythm pathway (Supplementary Fig. S4).

### Supplementation of granulosa cells with HS-EVs improved the viability and decreased the ROS accumulation

**Figure 9:**
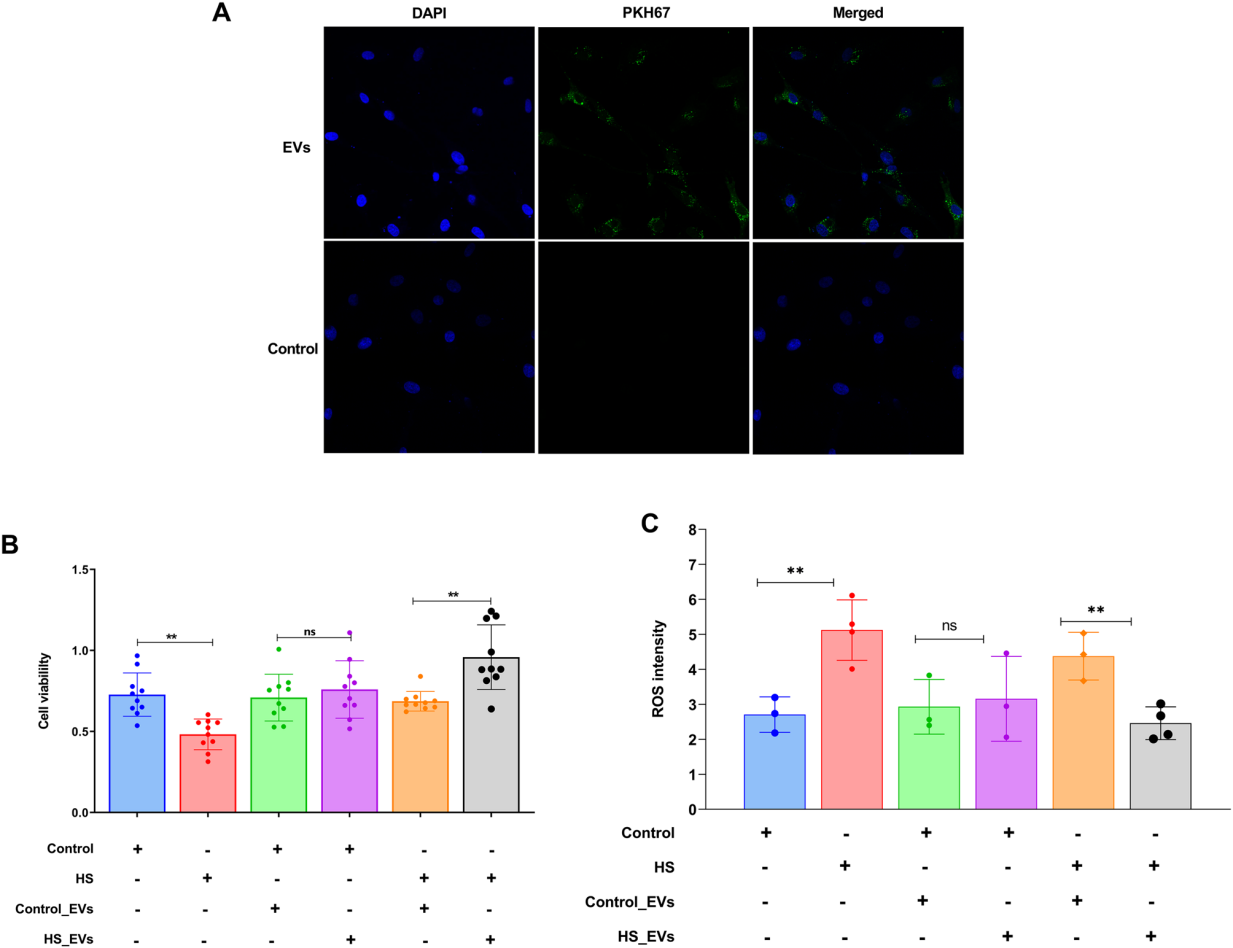
Uptake of EVs by granulosa cells under in vitro culture condition. Granulosa cells supplemented with PKH67-labelled EVs were up taken and internalized into the cytoplasm. The upper panels show cells supplemented with labeled EVs and the lower panels show cells supplemented with only PKH67 dye without EVs. The EVs are labeled in green fluorescence and the nucleus of the cells are stained in blue (DAPI) (**A**). Prior supplementation of HS-EVs to granulosa cells improved the viability upon exposure to HS (**B**) and the ROS accumulation was significantly reduced (**C**). The viability and ROS intensity data are presented as mean ± SEM and the mean differences were analyzed using the Two-tail student’s *t* test. **p < 0.01, *ns* not significant.

To analyze the impact of EV supplementation in delivering molecular signals that could protect cells from subsequent HS, we first examined the uptake of the in vitro supplemented EVs. For this, EVs were labeled with PKH67 membrane specific dye and co-incubated with granulosa cells. Twenty-four hours later, we examined the uptake of the labeled EVs in the cells using a confocal microscope. We were able to visualize labeled EVs in the cytoplasm of the granulosa cells and no signal of labeled EVs in the untreated control group (Fig. 9A). Granulosa cells supplemented with either the HS-EVs or Control-EVs were incubated at 37 °C for the first 24 h until sub-confluency is attained. Following this, half of the cells were subjected to HS, while the remaining were left at 37 °C and the impact of the EVs supplementation on the granulosa cells viability was examined. It was shown that granulosa cells without EVs supplementation subjected to HS have lower viability compared to those kept under 37 °C. Supplementation of either HS- or control-EVs has no measurable impact on the viability of cells when the cells were incubated under 37 °C. Interestingly, supplementation of HS-EVs improved the viability only in cells subsequently exposed to HS (Fig. 9B). This trend was consistent with the ROS accumulation assessment following EVs supplementation. It was shown that granulosa cells exposed to HS without any supplementation of EVs had a higher accumulation of intracellular ROS compared to the cells incubated at 37 °C. There were no measurable differences in the amount of ROS accumulation in granulosa cells supplemented with either HS- or control-EVs when the cells were incubated at 37 °C. However, when cells were supplemented with HS-EVs and subsequently exposed to HS, the ROS accumulation was significantly reduced compared to the cells supplemented with control-EVs (Fig. 9C).

### Supplementation of granulosa cells with HS-EVs shuttled protective messages against subsequent HS

**Figure 10:**
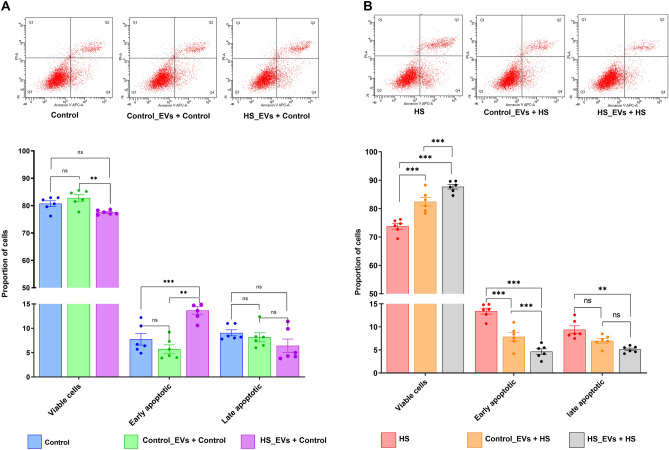
Effect of EVs supplementation on the level of granulosa cells apoptosis. Supplementation of HS-EVs showed to have deleterious effect granulosa cells by decreasing the proportion of viable cells and increasing the proportion of early apoptotic cells when the cells were cultured at 37 °C (**A**). However, supplementation of HS-EVs showed to have a beneficial effect by increasing the proportion of viable cells and decrease the proportion of early and late apoptotic cells when the cells were subsequently exposed to HS (**B**). The upper panels show the distribution of cells stained for both Annexin V and PI across the different quadrants. Data are presented as mean ± SEM and the mean differences were analyzed using one-way ANOVA followed by Bonferroni’s Multiple Comparison Test. **p < 0.01; ***p < 0.001, *ns* not significant.

To determine the protective signal of EVs against subsequent HS that could be shuttled to recipient cells, granulosa cells supplemented with either HS- or control-EVs were stained with annexin V/PI. It was shown that supplementation of cells with HS-EVs and subsequent incubation at 37 °C resulted in a significant reduction in the proportion of viable cells and an increase in the proportion of early apoptotic cells compared to cells supplemented with Control-EVs cultured at 37 °C (Fig. 10A). Contrary to this, cells supplemented with HS-EVs showed a significant increase in the proportion of viable cells and a significant reduction in the proportion of early and late apoptotic cells upon subsequent exposure to HS compared to the cells supplemented with control-EVs and without EV supplementation (Fig. 10B).

### EVs supplementation altered the expression of stress-associated transcripts

**Figure 11:**
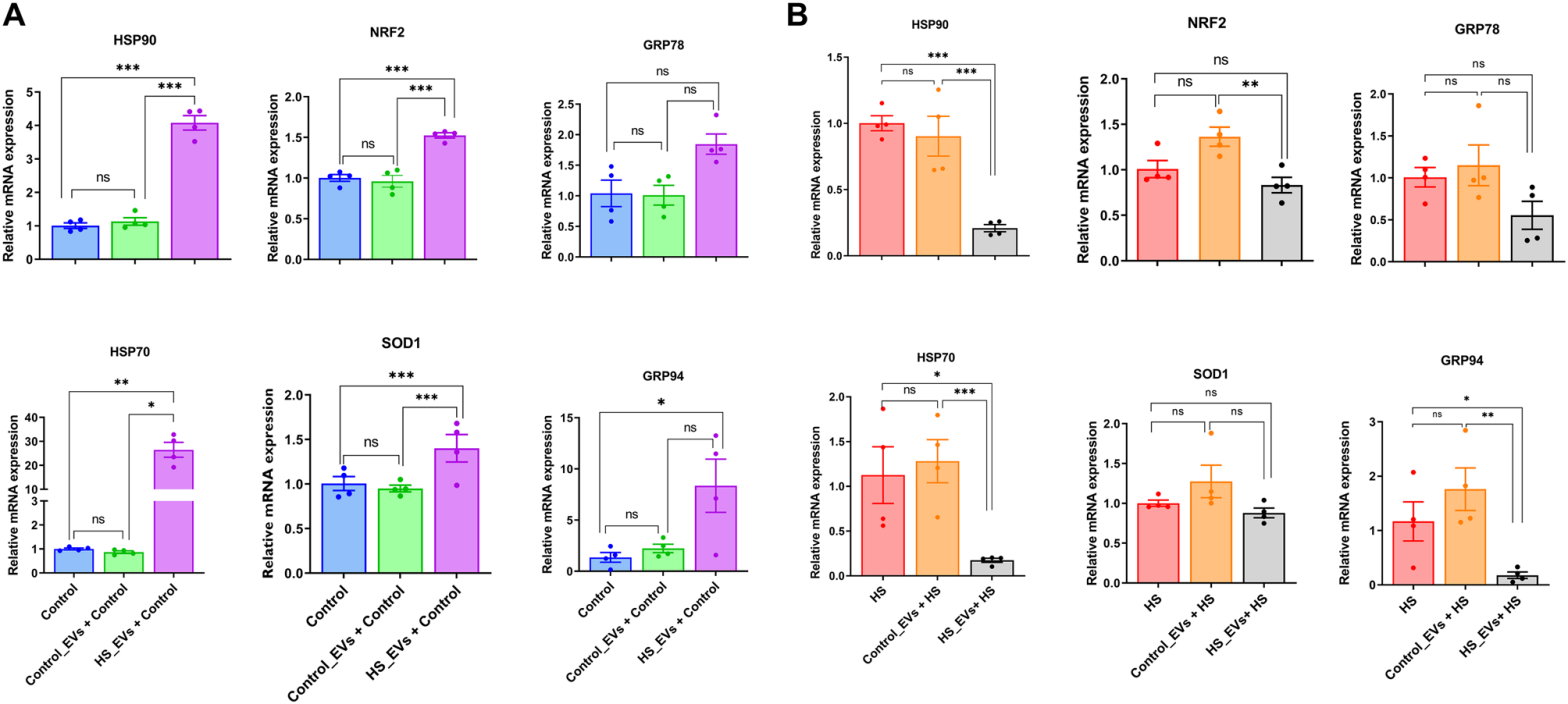
The impact of HS-EVs supplementation on the expression of stress-associated genes. The expression of the stress-associated was significantly increased in granulosa cells supplemented with HS-EVs and incubated at 37 °C (**A**) and the expression of these genes was significantly increased when the HS-EVs supplemented granulosa cells are exposed to subsequent HS (**B**). Data are presented as mean ± SEM and the mean differences were analyzed using one-way ANOVA followed by Bonferroni’s Multiple Comparison Test. *p < 0.05; **p < 0.01, ***p < 0.001, *ns* not significant.

After observing the protective effect of HS-EVs supplementation against subsequent HS, we examined the expression pattern of the stress-associated transcripts in granulosa cells supplemented with HS-EVs or control-EVs. Results showed that the expression pattern of HSP90, HSP70, NRF2, and SOD1 were enriched in granulosa cells supplemented with HS-EVs kept at 37 °C (Fig. 11A). Similarly, the expression of GRP78 and GRP94 tend to be higher in the same group, but not statistically significant (Fig. 11A). Contrary to this, the expression of these stress-associated transcripts was significantly downregulated in granulosa cells supplemented with HS-EVs and subsequently cultured under HS (Fig. 11B). There was no measurable difference in the expression of these transcripts between cells supplemented with control-EVs and those without EVs supplementation, irrespective of their exposure to HS.

## Discussion

The increase in the summer temperature is one of the leading causes of fertility impairment and a reduction in the performance of dairy cattle^2^. It is reported that HS affects various aspects of female fertility including ovarian function, oocyte maturation, and embryonic development^41^. The effect of HS on follicular development is associated with the reduction in the aromatase activity of granulosa cells and reduced estradiol concentration in the follicular fluid of dominant follicle^42^. In the present study, we used an in vitro granulosa cell culture model to decipher the bioactive molecular contents, mainly miRNAs and some known HS-associated transcripts, in EVs obtained from bovine granulosa in response to HS. Moreover, the protective potential of these EVs was examined by supplementation of HS-EVs in in-vitro cultured granulosa cells followed by subsequent exposure to HS.

Several studies have reported the transcriptional changes in granulosa cells in response to HS. For instance, the HSP70 HSP90, HSP60, and HSP27 were reported to increase in their synthesis in response to HS, which helps survival by maintaining the cellular hemostasis^43^. Genes involved in the stress response pathway and activation of transcription factors associated with the oxidative stress and heat shock factors (HSFs) have been reported in response to HS^44^. In agreement with this, our result showed that granulosa cells subjected to HS exhibited transcriptional activation of key genes involved in HS response (HSP70 and HSP90), oxidative stress (NRF2 and SOD1) and the endoplasmic reticulum stress (GRP78 and GRP94). Previously, we have reported that the expression of the stress-associated transcripts was significantly higher during the first 24 h post-HS exposure and was reversed at 48 h post-exposure^16^. This signifies the sharp increment of the HSPs help granulosa cells to develop adaptive response against chronic HS in an extended period. The HSP70 is one of the HSPs, which is rapidly induced in response to HS exposure^45^ and has been associated with induction of HS in mouse^17^ and porcine^46^ granulosa cells. Similarly, the elevation of HSP90 due to HS could be associated with the reduction of apoptosis by modulating the caspase signaling pathway^47^. Granulosa cells subjected to HS showed a significantly higher accumulation of ROS and increased total protein oxidation and decrease the viability. The significant accumulation of ROS, reduction of cell viability and increment in the early apoptotic cells in granulosa cells subjected to HS signifies the fact that HS induced oxidative stress in the targeted cells, as it is reported in previous studies^16,48^, that HS can activate the oxidative stress pathway and promote the generation of ROS in cells^49^. Accordingly, the negative impact of the HS-induced oxidative stress could be reversed by the supplementation of an antioxidant melatonin^50^. Moreover, the induction of oxidative stress due to HS is also reported to increase in the amount of total oxidized proteins, which could be due to the ROS-mediated cleavage of the peptide bonds or the incorporation of the carbonyl group into the protein of cells exposed to stress^51^.

Following the confirmation of the induction of HS, we examined the EVs released from granulosa cells subjected to HS. The size range distribution, the shape, the presence of the protein marker, and the RNA electropherogram of the EVs were in agreement with the typical values indicated by ISEV^52^. Interestingly, heat-stressed granulosa cells released significantly a greater number of EVs compared to the unstressed counterparts. Similar results were reported in which a large quantity of EVs was secreted from B cells and Jurka cells after exposure to HS^53,54^. The expression of the stress-related transcripts was quantified in the EVs released from stressed and unstressed cells and it was shown that HSP90 and SOD1 were significantly enriched in EVs released from stressed cells. Moreover, the expression of HSP70 and GRP94 tend to be higher in the EVs from stressed cells. Previous studies showed the enrichment of HSP90 and HSP70 in the EVs released from granulosa cells following exposure to HS^53^ and NRF2 and its downstream antioxidants, CAT and TXN1 following H_2_O_2_ treatment^32^. This signifies the fact that exposure of cells to HS not only induce the release of a large number of EVs into the extracellular space but also the release of stress-associated molecular signals as a response to elevated temperature. However, the regulatory mechanism behind the selection and loading of these specific molecules into EVs and its contribution to the cellular survival of the donor and recipient cells under HS needs further investigation.

In recent years, miRNAs have been implicated as important modifiers of gene expression and involved in wide ranges of reproductive pathophysiological conditions. Genome-wide miRNA expression profiling of granulosa cells and the corresponding released EVs showed a distinct pattern according to the treatment groups. EVs released from HS granulosa cells are enriched with bta-miR-1246, which also has the highest copy number compared to the other differentially expressed miRNAs. Previously, bta-miR-1246 was reported to be significantly upregulated in the serum of dairy cows exposed to HS and is predicted to be involved in mediating the oxidative stress and innate immunity response pathway^55^. Moreover, bta-miR-1246 was significantly upregulated in plasma of feedlot cattle exposed to high temperature and it was one of the highly enriched miRNAs in the circulation of the HS cattle^56^. In the same study, an in vitro functional analysis of miR-1246 showed its involvement in preventing apoptosis upon its overexpression by suppressing the expression of apoptosis-related genes; PCBP2 and CREBL2^56^. This implies that the bta-miR-1246 could be a promising candidate with anti-HS function in cattle that can be readily examined from the circulation. We also showed that the expression of miR-181a tends to be higher in EVs released from HS granulosa cells (Supplementary Table S3). Similarly, the expression of bta-miR-181a was significantly higher in the serum of HS cows^55^. The bta-miR-181a is known to be involved in various cellular biological processes including cell proliferation and apoptosis^57^. The impact of bta-miR-181a in peripheral blood mononuclear cells (PBMC) subjected to HS showed that inhibiting the miRNA would reverse the HS damage and improve the viability of PBMCs in Holstein cows^58^. The involvement of miRNAs in stress response is well known and EV-coupled miRNAs are differentially expressed in response to metabolic stress^30^. This implies the fact that the mechanism by which EVs pose protective signal could be by shuttling miRNAs preferentially enriched in the EVs obtained from stressed cells. Even though both the bta-miR-1246 and bta-miR-181a are enriched in the circulation of cows exposed to HS, they showed to have an antagonistic impact upon in vitro functional analysis. It is worth noting that the composition of miRNAs in the EVs does not necessarily have the same impact in the recipient cells, when they are individually evaluated under in vitro conditions.

EVs are implicated in mediating cell-to-cell communication and their greater advantage is the fact that a large number of molecules can be encapsulated irrespective of their smaller size and volume, which exposes the recipient cells to a multitude of molecules simultaneously, making it more efficient mechanism of molecule delivery^59^. In addition to that, the heterogeneity of the bioactive molecule encapsulated in the vesicles (RNA, proteins, and lipids) could also give a synergistic effect in the delivered messages to recipient cells. The stability of the EVs is another advantage, which enables them to travel long distances without releasing the encapsulated bioactive molecules^34^. To understand the role of EVs in cell-to-cell communication in the follicular microenvironment, EVs in follicular fluid are widely studied. Considering the dynamic nature of the follicular microenvironment, EVs in the follicular fluid could be originated from various follicular cells. Tracing follicular cells specific EVs with a distinct molecular signature in response to stress is hard to elucidate under in vivo condition. Therefore, the use of an in vitro model in the present study enabled us to identify granulosa cells' specific molecular responses to direct elevated culture temperature.

To determine the protective impact of the EVs released from HS granulosa cells, we primed granulosa cells with HS-EVs and subsequently exposed them to HS. It was shown that priming cells with HS-EVs make the cells resistant to subsequent HS and improved cell viability and reduced ROS accumulation, apoptosis rate, and protein oxidation. More interestingly, the abundance of the stress-associated transcripts was reduced in cells supplemented with HS-EVs and subsequently exposed to HS. This could imply the fact that granulosa cells population subjected to HS could release EVs that might cause an adaptive response in the recipient cells when subsequently exposed to the same stress conditions. However, whether those adaptive responses are stress type-specific or can also induce protection for any other stressors is an open question, which deserves future investigation. However, when cells were primed with HS-EVs but kept under normal temperature, the EVs could pose deleterious effects. This implies that the transmission of the molecular messages within the EVs and attaining phenotypic change in the recipient cells could depend on the stress condition of the recipient cells. The protective role of EVs released from cancer cells treated with ionizing radiation^60,61^ and HS^48^ was previously reported. Cells previously primed with HS-EVs showed a significant reduction in apoptosis and DNA damage compared to the cells treated with EVs obtained from unstressed cells^48^. This could be because of the fact that HS-EVs could carry molecules that help the recipient cells to develop immunity against subsequent stress. This was further validated by chemically inhibiting the EVs uptake pathway, in which cells treated with EVs-uptake inhibitors had a lower survival rate after exposure to HS.

Taken together, the results of the present study demonstrate that exposure of bovine granulosa cells to elevated temperature caused higher accumulation of intracellular ROS, induces apoptosis, facilitated oxidation of total protein. Moreover, the expression of HSPs and transcripts associated with oxidative and endoplasmic reticulum stresses were activated. Importantly, the expression profile of cellular and EV-coupled miRNAs can distinctly separate the cells and the corresponding EVs according to the stress exposure. In addition, based on previous studies and data from the present experiment, bta-miR-1246 can be a potential cellular and extracellular indicator of HS in bovine granulosa cells. The EVs released from the HS granulosa cells have the ability to induce a protective effect against subsequent stress by reducing the amount of ROS accumulation, increasing the viability of cells, and decreasing the proportion of early apoptotic cells. Moreover, the EVs released from HS granulosa cells are enriched with known stress-associated transcripts and miRNAs, which in turn support the notion of shuttling RNA molecules to induce an adaptive response in the recipient cells. It is worth noting that the priming of granulosa cells with HS-EVs could have a positive impact to protect cells from subsequent HS and potentially against other types of environmental and physiological stressors.

## Materials and methods

### Ovarian sample collection and isolation of granulosa cells

The collection of ovarian samples and aspiration of the granulosa cells was performed according to protocols previously described in^62,63^. Briefly, ovarian samples were obtained from a local slaughterhouse and transported in a thermos flask containing warm (37 °C) physiological saline solution (0.9% NaCl). Upon arrival, ovarian samples were repeatedly washed with warm phosphate-buffered saline without Ca^2+^/Mg^2+^ (PBS^−^). Ovaries were rinsed with 70% ethanol and washed again with warm PBS^−^. Granulosa cells were aspirated as previously described in^62^, from small and healthy growing follicles (3–6 mm in diameter) using 18 gauge needle fitted to 5 ml syringe. Aspirated follicular fluid was transferred into a 15 ml tube. The cumulus-oocyte-complexes were settled at the bottom of the tube and the follicular fluid with floating granulosa cells was transferred into a new tube. Following this, tubes containing the follicular fluid and granulosa cells were centrifuged at 700×*g* for 7 min and the supernatant was removed and the cell pellet was resuspended in 500 µl RBC lysis buffer for 1 min and washed twice with Dulbecco Modified Eagle Medium (DMEM)/F-12 Ham cell culture medium. Following this, the viability of cells was assessed using the Trypan-blue exclusion approach.

### Granulosa cells culture and induction of heat stress

Granulosa cells were cultured in tissue culture-treated 24-well plate as previously described^62,63^. Briefly, 2 × 10^5^ cells per well were seeded in 600 µl of medium (DMEM/F-12 Ham supplemented with 10% exosome-depleted FBS, 1% penicillin–streptomycin (Sigma-Aldrich), and 1% fungizone (Sigma-Aldrich). Cells were incubated at 37 °C in a humidified incubator with a 5% CO_2_ atmosphere for 24 h until sub-confluency is attained. Following this, half of the plates were kept at 37 °C (control) and the other half was incubated at 42 °C [heat-stressed (HS)] for another 24 h. Following this, the spent culture media were collected for EVs isolation, and the cells were harvested using 0.25% Trypsin–EDTA (Sigma-Aldrich) for further analysis.

### Analysis of the intracellular ROS accumulation

The impact of HS on granulosa cells intracellular ROS accumulation was determined as previously discussed in^16,63^. Briefly, 1 × 10^4^ cells were seeded in 96-well plates containing 100 µl of medium. After sub-confluency, the media was discarded and cells from both control and HS groups were stained using 10 µM fluorescent probe 6-carboxy-2′,7′-dichlorodihydrofluorescein diacetate (H_2_DCFDA) (Invitrogen, USA) for 30 min at 37 °C in the dark. Cells were washed twice with PBS and images were immediately captured using Leica DM IRB inverted fluorescence microscope (Leica, Germany) using a green fluorescence filter. Images were analyzed using ImageJ software (National Institutes of Health, USA).

### Cell viability assay

The impact of HS on the viability of granulosa cells was evaluated as previously described in^63^. Briefly, 1 × 10^4^ cells were seeded in 96-well plate containing 100 µl of the medium from both the control and HS groups Following this, 10 µl of CCK-8 (Dojindo Molecular Technology, Japan) was added into each well of cells from both groups and cells were incubated for 3 h at 37 °C in 5% CO_2_ atmosphere. Following this, the optical density (OD) of the formazan dye, which is positively correlated with the number of live cells, was measured at a wavelength of 450 nm in a microplate reader (BioTek Instruments, Germany). Reading from wells with only media without cells was used as blank for background correction.

### Annexin V/propidium Iodide (PI) staining

To determine the impact of HS on the level of apoptosis of the granulosa cells and to further distinguish the live or dead cells, annexin V/PI staining was performed. Briefly, cells were combinedly stained with annexin V-APC and PI using Cell Meter APC-Annexin V Binding Apoptosis Assay Kit (Biomol, USA), according to the manufacturer’s recommendations with modifications as mentioned in^64^. Cells were analyzed with LSRFortessa Flow cytometer (BD Biosciences, USA) and data were analyzed using FACSDiva 6.1.3 software (Becton Dickinson).

### Western blotting and analysis of total oxidized protein

The impact of HS on the abundance of the HSPs was analyzed using western blot analysis. For this, whole-cell protein lysates were prepared from cultured bovine granulosa cells using 1 × passive lysis buffer (Promega, USA). Following this, 30 µg of protein were run on 10–12% gradient SDS–polyacrylamide gel (SDS-PAGE) and transferred onto a nitrocellulose membrane. Membranes were blocked using 1 × Roti-block solution (Carl Roth, Germany) for 1 h under room temperature and incubated with diluted primary antibody of anti-HSP70 goat polyclonal antibody (1:200; Santa Cruz Biotechnology, Germany), anti-HSP90 mouse monoclonal antibody (1:200; Santa Cruz Biotechnology, Germany) and anti-ß-Actin mouse monoclonal antibody (1:500; Santa Cruz Biotechnology, Germany) overnight at 4 °C. Membranes were washed with 1 × TBST solution and incubated with horseradish peroxidase (HRP) conjugated donkey anti-goat (1:5000) or goat-anti-mouse goat (1:5000) secondary antibodies (Santa Cruz Biotechnology, Germany) for 2 h at room temperature. After washing the membranes with 1 × TBST, signals were visualized with Clarity Western ECL Substrate (Bio-Rad, Germany). Images were acquired using a gel Doc XRS imaging system (Bio-Rad, Germany). The density of the protein bands was quantified using ImageJ 1.48 (National Institutes of Health, USA). The abundance of the HSPs was normalized against the abundance of the values of the ß-actin.

Similarly, the introduction of carbonyl groups (aldehydes and ketones) to protein side chains, which could be studied as a sign of oxidative stress and is also characterized by their stable and irreversible nature was analyzed. For this, Oxidized Protein Western Blot Detection Kit (ab178020) was used according to the manufacturer's instructions with minor modifications as described in^65^. Briefly, DNPH derivatization was carried out on equal amounts (5 μg) of proteins, for 10 min at room temperature. Following neutralization, the sample mixtures were separated by SDS-PAGE then transferred to nitrocellulose membranes. The nonspecific protein binding was blocked by incubation with 5% non-fat milk dissolved in PBS containing 0.1% Tween 20. Thereafter, the proteins were incubated overnight with a primary antibody (1:5000 dilution) specific for the 2,4-dinitrophenol group (DNP moiety of the proteins) and a peroxidase-labeled secondary antibody goat anti-rabbit (1:5000 dilution), appropriately diluted in blocking buffer, for 1 h each. After the blots were developed using an enhanced chemiluminescence reagent kit, the images were acquired using Gel Doc XRS + imaging system (Bio-Rad, Germany).

### Isolation of EVs from the spent cell culture medium

The spent culture media obtained from both the control and HS cells were subjected to centrifugation at 500×*g* to remove the cells followed by 4000×*g* at 4 °C to remove the cellular debris and filtered through 0.22 µm to remove larger particles (200 nm). Following that, samples were centrifuged at 25,000×*g* for 30 min at 4 °C to remove the microvesicles. The purified cell culture medium was subjected to EVs isolation using two techniques. For large scale miRNA profiling, 5 ml of purified spent culture media was added with 1 ml of ExoQuick-TC (System Biosciences, USA) and mixed very well and incubated overnight at 4 °C. Samples were centrifuged at 1500×*g* to obtain the EVs pellet. After washing the pellet with PBS, EVs were resuspended in 500 µl PBS. A 50 µl fraction of the EVs was aliquoted for molecular and morphological characterization and the remaining samples were stored in − 80 °C until further use.

For validation and co-incubation experiments, EVs were isolated using ultracentrifugation procedure as previously described in^32^. Briefly, 5 ml of purified spent culture media from both the control and HS groups were subjected to ultracentrifugation at 120,000×*g* for 70 min using the Beckman SWTi55 rotor. The EVs pellets were washed with PBS and then centrifuged at 120,000×*g* for 70 min in the same rotor type. Finally, EVs were resuspended in 500 µL of PBS and stored in − 80 °C until further analysis.

### Morphological and molecular characterization of EVs

The presence of EV specific protein (CD63) in the isolated EVs and absence of cell-specific marker protein, cytochrome C (CYCS) were verified using the immunoblotting technique as described in^28^. Briefly, 100 µL of isolated EVs were re-suspended in 50 μl 1 × RIPA buffer and kept in ice for 10 min. To extract the protein, EVs were centrifuged at 12,000×*g* for 30 min at 4 °C. An equal amount of protein (30 µg) was run in 10–12% gradient SDS-PAGE and transferred onto a nitrocellulose membrane. Membranes were then blocked with a 1 × blocking buffer (Carl-Roth, Germany) for 1 h under room temperature. Following this, membranes were incubated with anti-CD63 rabbit polyclonal (1:250 System Biosciences, USA). Anti-TSG101 rabbit polyclonal (1:250 System Biosciences, USA), Anti-Flotlliin 1 rabbit polyclonal (1:250 System Biosciences, USA) and anti-CYCS goat polyclonal (1:350 Santa Cruz Biotechnology, Germany) primary antibodies overnight at 4 °C. After washing the membranes with 1X TBST, they were incubated with a secondary antibody of either goat anti-rabbit (1:5000, Santa Cruz Biotechnology Inc, Germany) or donkey anti-goat (1:5000, Santa Cruz Biotechnology Inc, Germany) for 1 h at room temperature. The proteins bands were visualized using enhanced chemiluminescence substrate (Bio-Rad, Germany) and images were acquired using Gel Doc XRS + imaging system (Bio-Rad, Germany).

The size and concentration of isolated EVs were determined using the Nanoparticle tracking analysis (NTA). Briefly, 30 µl of purified EVs was diluted in 970 µl of PBS and assembled into the Nanosight LM10 microscope (Nanosight, Salisbury, UK) fitted with LM14C laser. For each sample, 5 independent video measurements were recorded, and video files were analyzed with NTA software version 3.2. Similarly, the morphology and size of the EVs were analyzed using a transmission electron microscope (TEM). For this, a drop of 30 µl purified EVs was placed on parafilm and the Formvar/carbon-coated grids were placed on top of the EVs drops and allowed to stand for 5 min to absorb the EVs. The grids with adherent EVs were washed in drops of ddH_2_O and fixed 30 µL of 2% uranyl acetate and examined under an electron microscope. All EV-related data of the experiments conducted on bovine granulosa cells have been submitted to the EV-TRACK knowledgebase (https://evtrack.org) under the EV-TRACK ID: EV200016^66^.

### Total RNA isolation and quality control

Total RNA enriched with miRNAs was isolated from granulosa cells and the purified EVs using miRNeasy mini kit (Qiagen, Germany) and Norgen exosomal RNA isolation kit (Norgen, Canada), respectively according to the manufacturers’ instruction. On column DNA digestion was performed to remove genomic DNA contaminants. The concentration and integrity of the isolated total RNA samples were assessed with NanoDrop 8000 spectrophotometer (NanoDrop Technologies, DE) and Agilent 2100 Bioanalyzer (Agilent Technologies, CA), respectively. Until further use, the isolated RNA samples were stored in − 80 °C.

### Quantification of stress-related genes using qRT-PCR

The relative abundance of selected stress-related genes (HSP90, HSP70, NRF2, SOD1, GRP94, and GRP78) was quantified in both granulosa cells and the corresponding EVs using qRT-PCR. For this, cDNA was synthesized by reverse transcribing from an equal amount of total RNA using the First-Strand cDNA synthesis kit (Thermo Fisher Scientific) with oligo(dT)18 primers. Primers for each gene were designed using the Primer-Blast (https://www.ncbi.nlm.nih.gov/tools/primer-blast/). Quantification of each transcript was performed in quadruplicates of 20 µl of reaction volume on the same plate containing 0.3 μl of each primer, 10 μl of 1 × SYBR Green I Master Mix (Bio-Rad), and 2 μl of cDNA template and 7.4 μl of ddH_2_O. A negative control containing 2 μl of ddH_2_O instead of cDNA was parallelly run for each transcript. The following thermocycling conditions were applied for amplification: initial denaturation at 95 °C for 3 min, followed by 40 cycles of amplification at 95 °C for 15 s, and 60 °C for 60 s. The specificity of the amplification was determined by melting curve analysis generated at the end of each run. The geometric mean of the expression of GAPDH and ß-Actin was used as an internal control to normalize the expression of genes of interest. The relative abundance of each transcript was analyzed using the comparative CT (^ΔΔ^CT) method^67^. The list of primers used is indicated in supplementary table S1.

### Library preparation and next-generation sequencing

The preparation of the miRNA library was done using the QIAseq miRNA Library Kit (Qiagen, Germany). For this, a total of 100 ng (from the granulosa cells) and 50 ng (from EVs) of total RNA were converted into miRNA NGS library. After ligating adapters with unique molecular identifiers (UMI) to the 5′ and 3′ ends of the RNAs, cDNA was synthesized using reverse transcription (RT) and during the RT reactions, unique PCR indices were added. Following that a clean-up of the cDNA was performed using the Qiabeads magnetic beads (Qiagen, Germany), and the cDNA samples were amplified (16 cycles) using the indexing forward and universal reverse primers. Following the library amplification, a clean-up of the library was performed with Qiabeads magnetic beads (Qiagen, Germany). Library quality control was performed using Bioanalyzer 2100 (Agilent). Based on the quality of the insert and concentration, libraries were pooled in equimolar ratios and pooled libraries were quantified using qPCR. Pooled libraries were sequenced on a NextSeq550 sequencing platform (Illumina, CA) according to the manufacturer instructions. On average, 14 million Single-end reads were generated per samples with a read length of 75 nt. Raw data files were de-multiplexed and the FASTQ file of each sample was generated using the bcl2fastq software (Illumina, CA).

### Sequence quality assessment and processing

The quality of the raw FASTQ files was assessed using the FASQC (0.11.7) (https://www.bioinformatics.babraham.ac.uk/projects/fastqc). The per base and average sequence quality were thoroughly inspected and every read with an average quality score of 30 and above was of higher quality. Information about the adaptors and UMI in the raw reads was extracted using Cutadapt (1.11) (https://cutadapt.readthedocs.io/en/stable/). Adaptors attached to each read were trimmed and reads with identical UMI were collapsed into a single read. The raw FASTQ files and processed CSV files of the granulosa cells and EVs have been deposited in NCBI's Gene Expression Omnibus and are accessible through GEO Series accession numbers of GSE146490 and GSE146491, respectively.

### Mapping and annotation of Bovine miRNAs

Quality reads were mapped against the *Bos taurus* reference genome release 72 (https://ftp.ensemblorg.ebi.ac.uk/pub/release-72/fasta/bos_taurus/dna/) using Bowtie2 (2.2.2)^68^ and no more than one mismatch was tolerated in the first 32 bases of the sequence read. Mapped sequence reads were mapped against bovine matured and precursor miRNAs annotated in miRbase (release 22) (https://www.mirbase.org/).

### Normalization and differential expression of miRNAs

Raw miRNA expression value was normalized using the trimmed mean of M-values normalization method (TMM normalization)^69^. This normalization technique compensates for sample-specific effects caused by the variation in library size/sequencing depth between samples. Moreover, it compensates potential under- and over-sampling effects by trimming and applying scaling factors that minimize log-fold changes between samples across most of the miRNAs. Differential expression analysis was performed using TMM in the EdgeR statistical software package (Bioconductor, https://www.bioconductor.org/).

### Target gene prediction and ontological classification

Genes targeted by the differentially expressed miRNAs were identified using the TargetScan analysis tool (Release 7.2, https://www.targetscan.org/) with a threshold of cumulative weighted context ++ score ≤  − 0.2^70^. The list of predicted target genes of up- or down-regulated miRNAs were submitted to the DAVID bioinformatics web-tool (https://david.abcc.ncifcrf.gov/) for ontological classification. Significant pathways were identified from the Kyoto Encyclopaedia of Genes and Genomes (KEGG) database^71^. Interaction networks of the targeted genes and the identified pathways were constructed by Cytoscape (https://www.cytoscape.org/) and ClueGO (https://apps.cytoscape.org/apps/cluego)^72,73^.

### Validation of differentially expressed miRNAs using droplet digital PCR

To validate the expression miRNAs differentially expressed in the NGS data, a ddPCR technique was used as described in^74^. Briefly, the copy numbers of the selected differentially expressed miRNAs were quantified in EVs and granulosa cells samples using TaqMan miRNA assays (Applied Biosystems, Foster City, CA, USA) and QuantaLife QX200 droplet digital PCR (ddPCR) system (Bio-Rad Inc.) according to the manufacturer’s instructions. Briefly, 10 ng of total RNA was reverse transcribed (RT) using a TaqMan MicroRNA Reverse Transcription Kit (Thermo Fisher Scientific, Waltham, MA, USA). The RT reaction mixtures with a total volume of 15 μl were prepared for each miRNA with 0.15 μl dNTPs (100 mM), 1.5 μl 10 × RT buffer, 0.19 μl RNase inhibitor (20 U/μl), 1 μl reverse transcriptase (50 U/μl), 3 μl miRNA-specific stem-loop primer and 10 ng of the RNA sample. The reaction mixture was incubated at 16 °C for 30 min then 42 °C for 30 min followed by 85 °C for 5 min. For miRNA quantification, 20 μl of ddPCR mixtures were prepared with 5 μl of the respective RT product, 1 μl TaqMan MicroRNA probe (FAM, 250 nM) and 10 μl ddPCR Supermix for Probes. Reaction mixtures were loaded into DG8 droplet generator cartridges with 70 μl of oil for the probes and subjected to a QuantaLife droplet generator. Droplets of each sample were carefully transferred to a 96-well PCR plate and amplified in a thermal cycler (C1000 Touch; Bio-Rad Inc.) using the following conditions: 95 °C for 10 min, 40 cycles of 94 °C for 30 s, 60 °C for 1 min, and 98 °C for 10 min. Samples were read in a QuantaLife QX200 Droplet Reader, and the absolute quantification of copy numbers of each miRNA was determined using QuantaSoft software (version 1.7). The expression of miRNAs was normalized to bta-miR-361, as it was the most stable expressed miRNA under normal and HS conditions.

### Labeling of EVs with PKH67 dye and cellular uptake

To verify the uptake of EVs by granulosa cells, 30 µl of EVs were mixed with 1 ml of diluent C containing 2 µl (2 µM) of PKH67 (Sigma-Aldrich), a green fluorescence dye that labels lipid membranes of cells^33^. The mix was left at room temperature for 10 min and the reaction was stopped by adding an equal volume of DMEM/F-12 media + 1% BSA for quenching and incubated at 37 °C for 30 min. Sterile PBS was incubated with 2 µl of PKH67 (Sigma-Aldrich) and considered as negative controls. Labeled EVs were washed twice to remove excess dye using ultracentrifugation at 120,000×*g* for 70 min using the Beckman SWTi55 rotor. Following this, granulosa cells were cultured in an 8-well chamber slide in DMEM/F-12 supplemented with 10% EVs depleted FBS and the labeled EVs. Twenty-four hours later, cells were fixed using 4% PFA and incubated at 4 °C overnight. Cells were washed twice with CMF and mounted in mounting medium containing DAPI (Dabco, Belgium). Images were captured Zeiss LSM-780 laser scanning confocal microscope (Carl Zeiss, Germany) and signals were analyzed with ImageJ 1.48v (National Institutes of Health, USA).

### Coincubation of EVs with granulosa cells and induction of heat stress

Prior to the coincubation of the HS-EVs and control-EVs with granulosa cells, a working concentration of DMEM/F-12 supplemented with either HS-EVs or control EVs was prepared as previously described in^33^. Briefly, EVs isolated from 12 ml of conditioned media were resuspended in 1.2 ml of DMEM/F-12 media. Following this, a working granulosa cell culture media composed of DMEM/F-12 + 10% exosome-depleted FBS + 1% penicillin–streptomycin (Sigma-Aldrich, Germany), and 1% fungizone (Sigma-Aldrich, Germany) + 10% of either HS-EVs or control-EVs was prepared. Granulosa cells were cultured in the prepared working media at 37 °C in a humidified incubator with 5% CO_2_ atmosphere for 24 h until sub-confluency was attained. Twenty-four hours later, half of the plates were transferred into another humidified incubator calibrated to 42 °C with 5% CO_2_ atmosphere. Following co-incubation for 24 h, cells were subjected to ROS accumulation, cell viability, annexin V/PI, gene expression, and total protein oxidation assays as described above. A schematic summary of the experimental design is illustrated in Supplementary Fig. S1A and B.

### Statistical analysis

Data were analyzed in GraphPad Prism version 8.1.0 (GraphPad, USA). Statistical difference in the mean values of the two groups was compared using the two-tailed Student’s *t* test. Moreover, Statistical differences between the mean values of more than two groups were compared using the One-way Analysis of Variance (ANOVA) followed by Bonferroni’s Multiple Comparison Test. Data are presented as Mean ± SEM of biological replicates. Statistical significance was set at *p* ≤ 0.05.

## Supplementary information

Supplementary Table S1.

Supplementary Table S2.

Supplementary Table S3.

Supplementary Table S4.

Supplementary figures.

## Data Availability

Sequence data files are available in GEO database with the accession numbers of GSE146490 and GSE146491. Similarly, All EV-related data have been submitted to the EV-TRACK knowledgebase and can be assessed under the EV-TRACK ID: EV200016.
