# Supplementary material for: Extracellular vesicles shuttle protective messages against heat stress in bovine granulosa cells
Source: Sci Rep. 2020 Sep 25;10:15824. doi: 10.1038/s41598-020-72706-z (PMC7519046; doi:10.1038/s41598-020-72706-z)
Supplement: Supplementary file 1 — Supplementary Table S1. [file 41598_2020_72706_MOESM1_ESM.docx]

| **Gene name** | **Accession number** | **Primer sequences** |
| --- | --- | --- |
| HSP70 | NM_001038505.2 | F5´aatgccagttgccaatgctg-3´  R5´atcgagagttcctccaccca-3´ |
| HSP90 | NM_001012670 | F5´tcactgaggaaatgccaccc-3´  R5´atggagacagagcgctgaac-3´ |
| GRP78 | NM_001075148 | F5´tgcgaagccctatagctgac-3´  R5´agtaggtggtacccaggtcg-3 |
| GRP94 | NM_174700 | F5´tgctgtgtggagagggaatg-3´  R5´tcctgtgaccacaatcccaa-3 |
| NRF2 | NM_001011678 | F5´cccagtcttcactgctcctc-3´  R5´aggcaggaactagcgaaag-3´ |
| SOD1 | NM_174615 | F5´agaggcatgttggagacctg-3´  R5´cagcgttgccagtctttgta-3´ |
| β-ACTIN | NM_173979 | F5´tgtccaccttccagcagat-3´  R5´tcaccttcaccgttccagt-3´ |
| GAPDH | NM_001034034 | F5´aatggagccatcaccatc  R5´gtggttcacgcccatcaca |

**Supplementary Table 1**. Sequence specific primers used for RT-PCR analysis
