# Supplementary material for: Extracellular vesicles shuttle protective messages against heat stress in bovine granulosa cells
Source: Sci Rep. 2020 Sep 25;10:15824. doi: 10.1038/s41598-020-72706-z (PMC7519046; doi:10.1038/s41598-020-72706-z)
Supplement: Supplementary file 2 — Supplementary Table S2. [file 41598_2020_72706_MOESM2_ESM.docx]

| Group | Sample  ID | QC passed reads | Genome- mapped reads | Genome-mapped reads  (%) | Mapped to annotated  miRNAs | Mapped  to annotated miRNAs  (%) |
| --- | --- | --- | --- | --- | --- | --- |
| Control | R1 | 15,594,302 | 7,592,640 | 48.69 | 2,222,447 | 14.25 |
|  | R2 | 14,086,548 | 3,325,583 | 23.61 | 5,437,451 | 38.60 |
|  | R3 | 11,346,591 | 2,789,442 | 24.58 | 4,083,860 | 35.99 |
| HS | R1 | 16,954,376 | 7,884,800 | 46.51 | 3,452,980 | 20.36 |
|  | R2 | 16,127,819 | 6,235,253 | 38.66 | 4,093,930 | 25.39 |
|  | R3 | 16,015,956 | 5,059,491 | 31.59 | 5,444,664 | 34.01 |
| Control_EVs | R1 | 7,271,963 | 1,621,033 | 22.29 | 161,409 | 2.22 |
|  | R2 | 5,465,926 | 964,106 | 17.64 | 416,307 | 7.62 |
|  | R3 | 3,949,622 | 792,553 | 20.07 | 153,172 | 3.88 |
| HS_EVs | R1 | 16,014,683 | 3,695,415 | 23.08 | 431,671 | 2.70 |
|  | R2 | 8,168,683 | 1,804,425 | 22.09 | 219,604 | 2.69 |
|  | R3 | 8,599,154 | 1,960,415 | 22.80 | 166,414 | 1.94 |

**Supplementary Table 2**: Summary of sequence reads alignment to bovine reference genome (UMD 3.1) and known miRNAs annotated in miRbase (miRbase_20)
