# Supplementary material for: Extracellular vesicles shuttle protective messages against heat stress in bovine granulosa cells
Source: Sci Rep. 2020 Sep 25;10:15824. doi: 10.1038/s41598-020-72706-z (PMC7519046; doi:10.1038/s41598-020-72706-z)
Supplement: Supplementary file 3 — Supplementary Table S3. [file 41598_2020_72706_MOESM3_ESM.docx]

| MicroRNA ID | Log_2_FC | P-value | FDR |
| --- | --- | --- | --- |
| bta-miR-10176-5p | 2.67 | 0.044395 | 0.873387 |
| bta-miR-2285cs | 2.49 | 0.016503 | 0.495511 |
| bta-miR-2449 | 2.15 | 0.001139 | 0.11517 |
| bta-miR-2389 | 1.80 | 0.013084 | 0.495511 |
| bta-miR-2397-3p | 1.80 | 0.035635 | 0.779422 |
| bta-miR-11987 | 1.61 | 0.00371 | 0.211864 |
| bta-miR-12057 | 1.56 | 0.018934 | 0.526045 |
| bta-miR-2400 | 1.43 | 0.015873 | 0.495511 |
| bta-miR-6523a | 1.41 | 3.11E-07 | 6.4E-05 |
| bta-miR-2448-3p | 1.34 | 0.000893 | 0.114707 |
| bta-miR-2448-5p | 1.30 | 0.009248 | 0.475372 |
| bta-miR-6775 | 1.29 | 0.016652 | 0.495511 |
| bta-miR-11980 | 1.22 | 0.003698 | 0.211864 |
| bta-miR-2285bd | 1.20 | 0.034768 | 0.776995 |
| bta-miR-2339 | 1.15 | 0.00239 | 0.16377 |
| bta-miR-149-5p | 1.08 | 0.015146 | 0.495511 |
| bta-miR-2447 | -1.07 | 0.001232 | 0.11517 |
| bta-miR-504 | -1.09 | 0.048273 | 0.902265 |
| bta-miR-12030 | -1.17 | 0.000162 | 0.027682 |
| bta-miR-184 | -1.22 | 0.012691 | 0.495511 |
| bta-miR-488 | -1.23 | 0.017227 | 0.495511 |
| bta-miR-2285ak-5p | -1.23 | 0.026641 | 0.667971 |
| bta-miR-11977 | -1.23 | 0.032266 | 0.774055 |
| bta-miR-2427 | -1.24 | 0.032378 | 0.774055 |
| bta-miR-2475 | -1.58 | 0.002114 | 0.156201 |
| bta-miR-6120-3p | -1.58 | 6.66E-15 | 6.85E-12 |
| bta-miR-2284m | -1.81 | 0.016443 | 0.495511 |
| bta-miR-6121-3p | -1.84 | 1.85E-08 | 4.76E-06 |
| bta-miR-2344 | -2.34 | 1.24E-11 | 6.39E-09 |
| bta-miR-2314 | -2.37 | 0.024171 | 0.653897 |
| bta-miR-2318 | -2.59 | 9.66E-09 | 3.31E-06 |
| bta-miR-2477 | -3.99 | 0.002127 | 0.156201 |

**Supplementary Table 3:** List of differentially expressed miRNAs in granulosa cells subjected to HS
