# Supplementary material for: Extracellular vesicles shuttle protective messages against heat stress in bovine granulosa cells
Source: Sci Rep. 2020 Sep 25;10:15824. doi: 10.1038/s41598-020-72706-z (PMC7519046; doi:10.1038/s41598-020-72706-z)
Supplement: Supplementary file 4 — Supplementary Table S4. [file 41598_2020_72706_MOESM4_ESM.docx]

| MicroRNA ID | Log_2_FC | P-value | Adjusted P-value |
| --- | --- | --- | --- |
| bta-miR-2904 | 5.11 | 6.27E-06 | 0.006446 |
| bta-miR-545-5p | 3.84 | 0.013413 | 0.999679 |
| bta-miR-2320-5p | 3.60 | 0.018698 | 0.999679 |
| bta-miR-27a-5p | 3.45 | 0.023492 | 0.999679 |
| bta-miR-500 | 2.92 | 0.041224 | 0.999679 |
| bta-miR-1246 | 2.87 | 0.00057 | 0.19539 |
| bta-miR-628 | 2.74 | 0.030009 | 0.999679 |
| bta-miR-11987 | 2.55 | 0.001439 | 0.318841 |
| bta-miR-374a | 2.12 | 0.000524 | 0.19539 |
| bta-miR-374b | 1.68 | 0.001551 | 0.318841 |
| bta-miR-491 | 1.56 | 0.004762 | 0.815805 |
| bta-miR-181a | 1.39 | 0.024216 | 0.999679 |
| bta-miR-29b | 1.33 | 0.006561 | 0.963591 |
| bta-miR-454 | 1.19 | 0.019381 | 0.999679 |
| bta-miR-23b-3p | 1.16 | 0.008501 | 0.999679 |
| bta-miR-150 | 1.02 | 0.015017 | 0.999679 |
| bta-miR-99b | 1.00 | 0.019184 | 0.999679 |
| bta-miR-432 | -1.05 | 0.023947 | 0.999679 |
| bta-miR-32 | -1.21 | 0.01174 | 0.999679 |
| bta-miR-145 | -1.22 | 0.048484 | 0.999679 |

**Supplementary Table 4**: List of differentially expressed miRNAs in EVs derived from granulosa cells subjected to HS
