# Supplementary material for: Extracellular vesicles shuttle protective messages against heat stress in bovine granulosa cells
Source: Sci Rep. 2020 Sep 25;10:15824. doi: 10.1038/s41598-020-72706-z (PMC7519046; doi:10.1038/s41598-020-72706-z)
Supplement: Supplementary file 5 — Supplementary figures. [file 41598_2020_72706_MOESM5_ESM.pdf]

Extracellular vesicles shuttle protective messages against heat stress in bovine granulosa cells

Samuel Gebremedhn, Ahmed Gad, Hoda Samir Aglan, Jozef Laurincik, Radek Prochazka, Dessie Salilew-Wondim, Michael Hoelker, Karl Schellander, Dawit Tesfaye

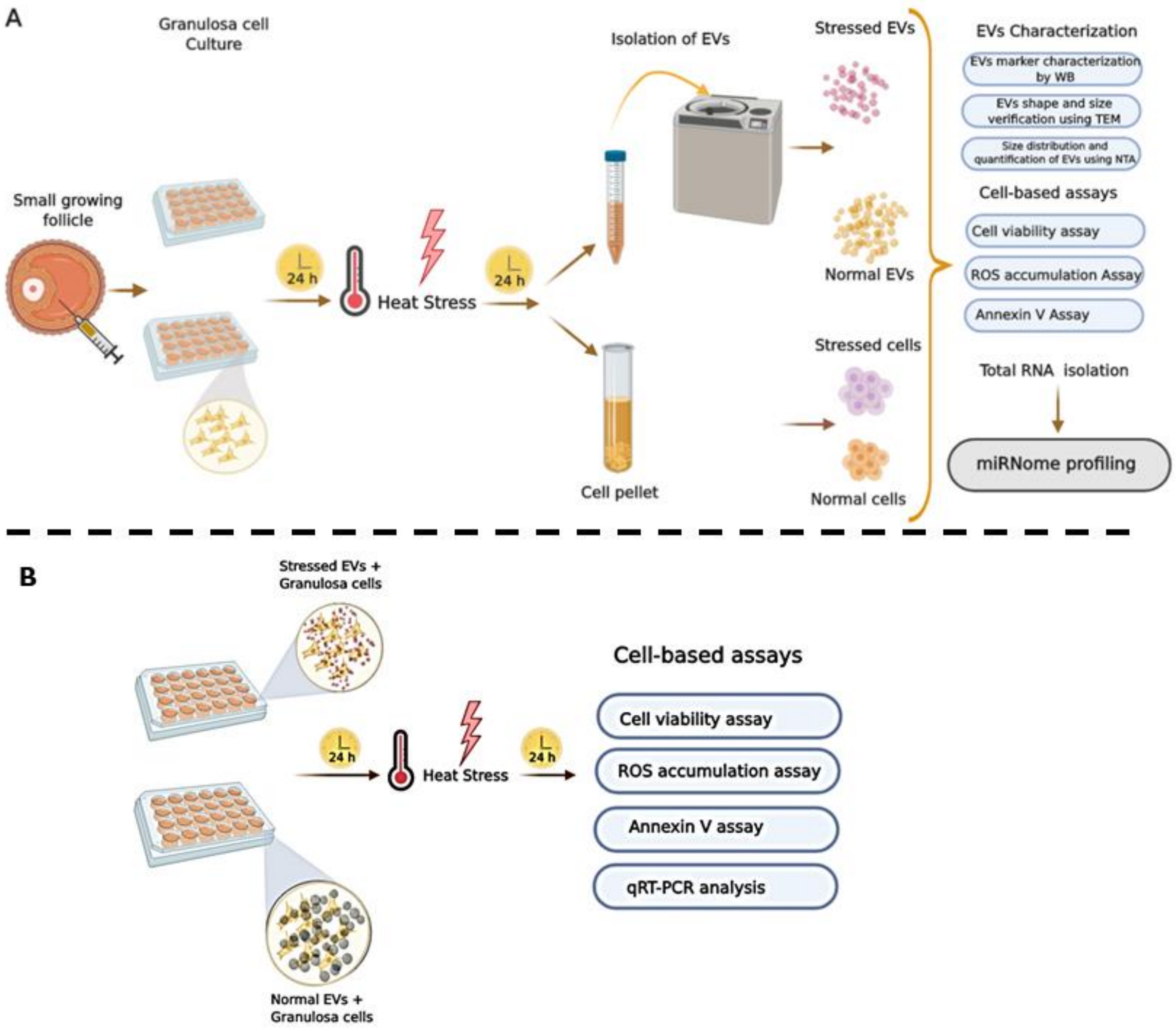

Supplementary Figure S1: Schematic illustration of the experimental design. Granulosa cells aspirated from small antral follicles cultured under in vitro condition were either exposed to HS or kept at 37ºC. The EVs were isolated using ultracentrifugation and cells were used for various phenotypic analyses. EVs were characterized using western blotting (WB), NTA, and TEM. Total RNA isolated from both the EVs and cells were subjected to genome-wide miRNAs profiling (**A**). Supplementation of granulosa cells with either HS-EVs or control EVs and 24 hours later, cells were incubated under 37ºC or 42ºC. Following this, the impact of the EVs supplementation was assessed using cell-based assays (**B**)

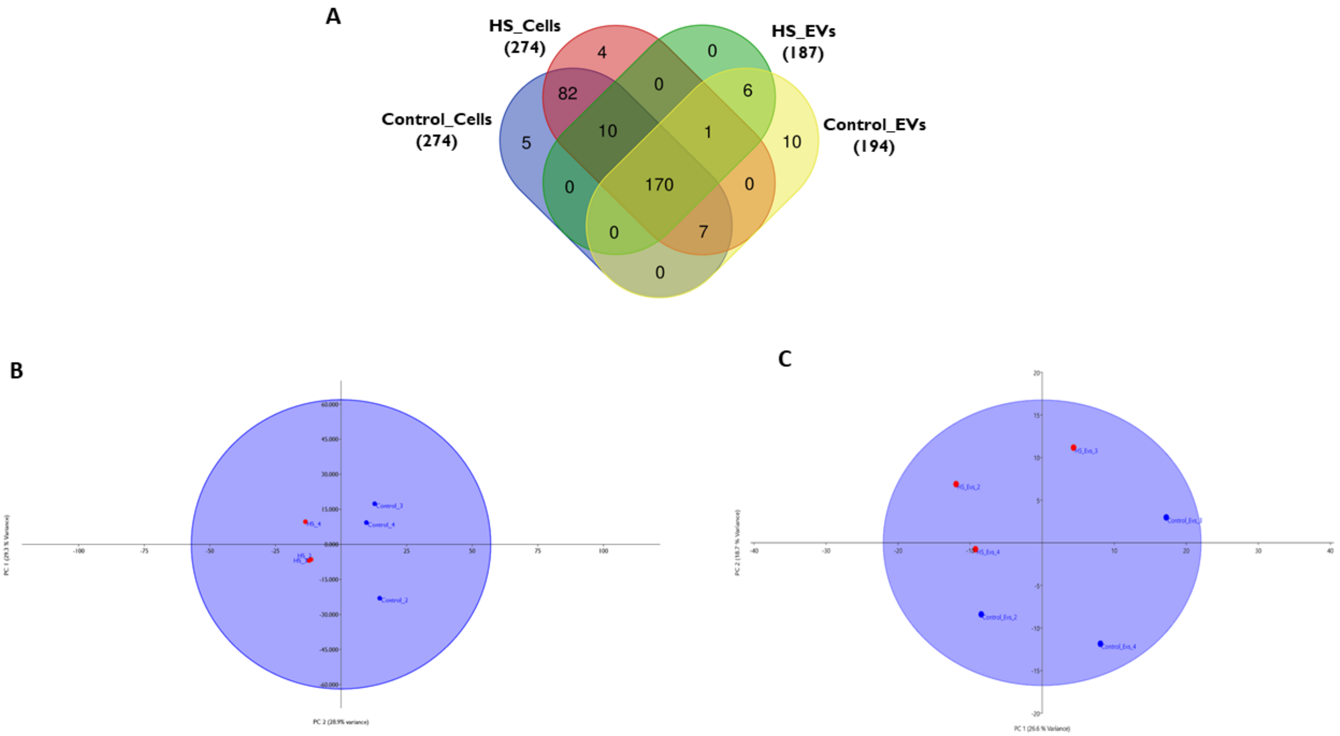

Supplementary Figure S2: Detection of mature miRNAs in granulosa cells cultured under different temperatures and the corresponding EVs. The number of commonly and uniquely detected miRNAs in granulosa cells and the EVs from the HS and control groups are indicated in the Venn diagram (**A**). Principal component analysis (PCA) in granulosa cells (**B**) and EVs (**C**) according to the expression of the miRNAs. The red dots represent the HS group and the blue dots represent the control group.

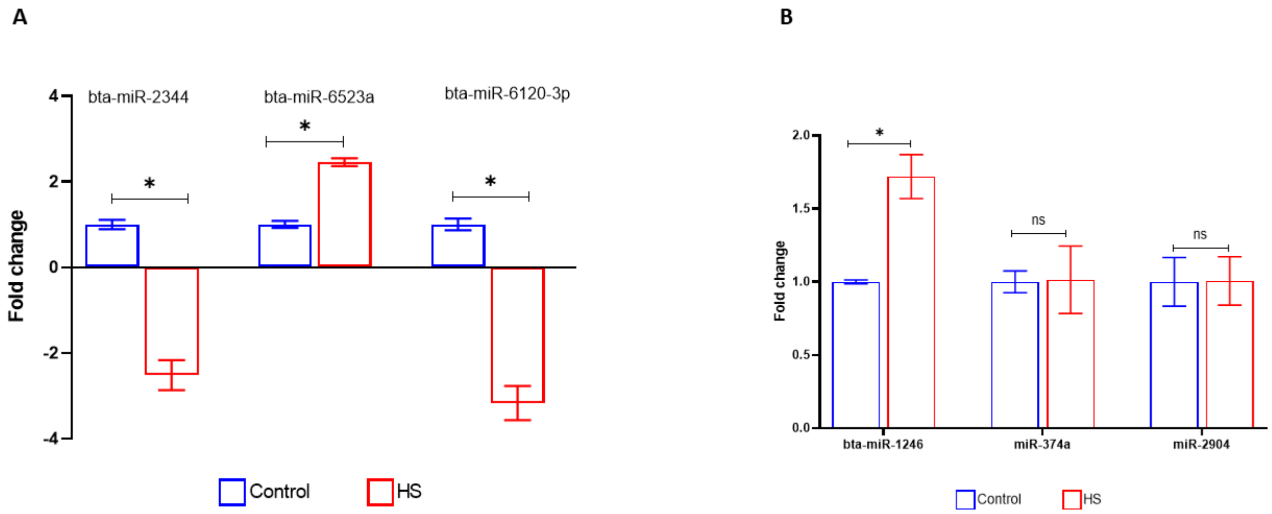

Supplementary Figure S3: Validation of the expression of differentially expressed miRNAs. The expression of miRNAs differentially expressed in granulosa cells (**A**) and EVs (**B**) subjected to HS was validated using droplet digital PCR (ddPCR). Data are presented as mean ± SEM and the mean differences were analyzed using the Two-tail student´s t-test. **: $p < 0.01$, ns: not significant.

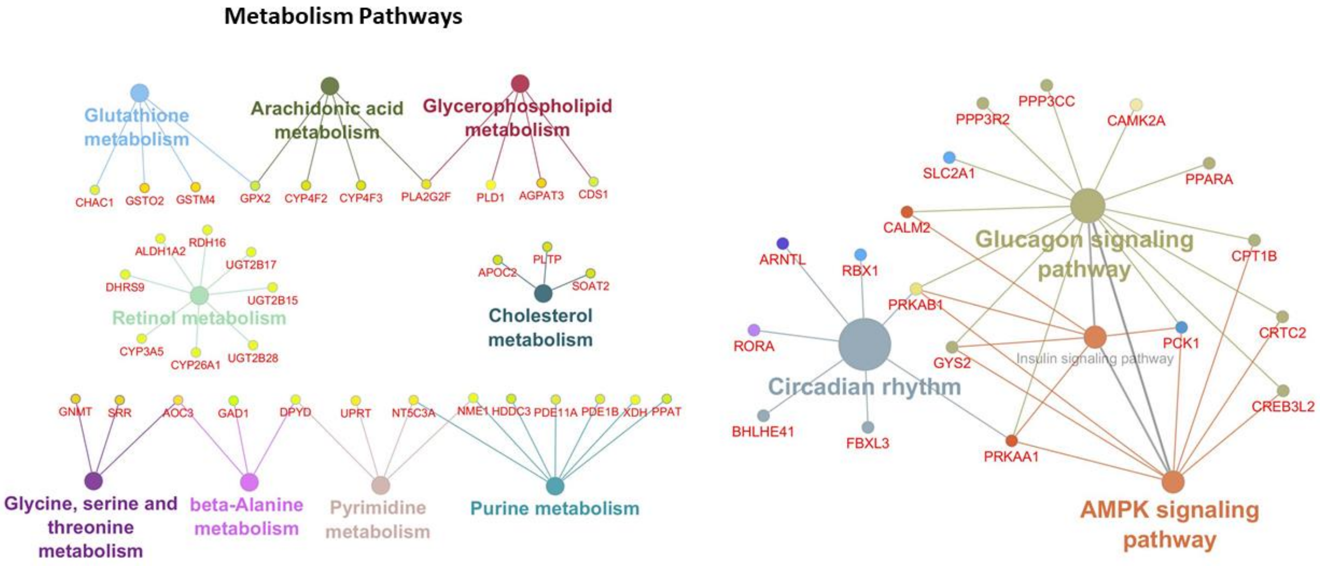

Supplementary Figure S4: Interaction networking between pathways. Signaling and metabolism pathways enriched by the predicted target genes of miRNAs, which were differentially expressed in EVs obtained from granulosa cells exposed to HS.

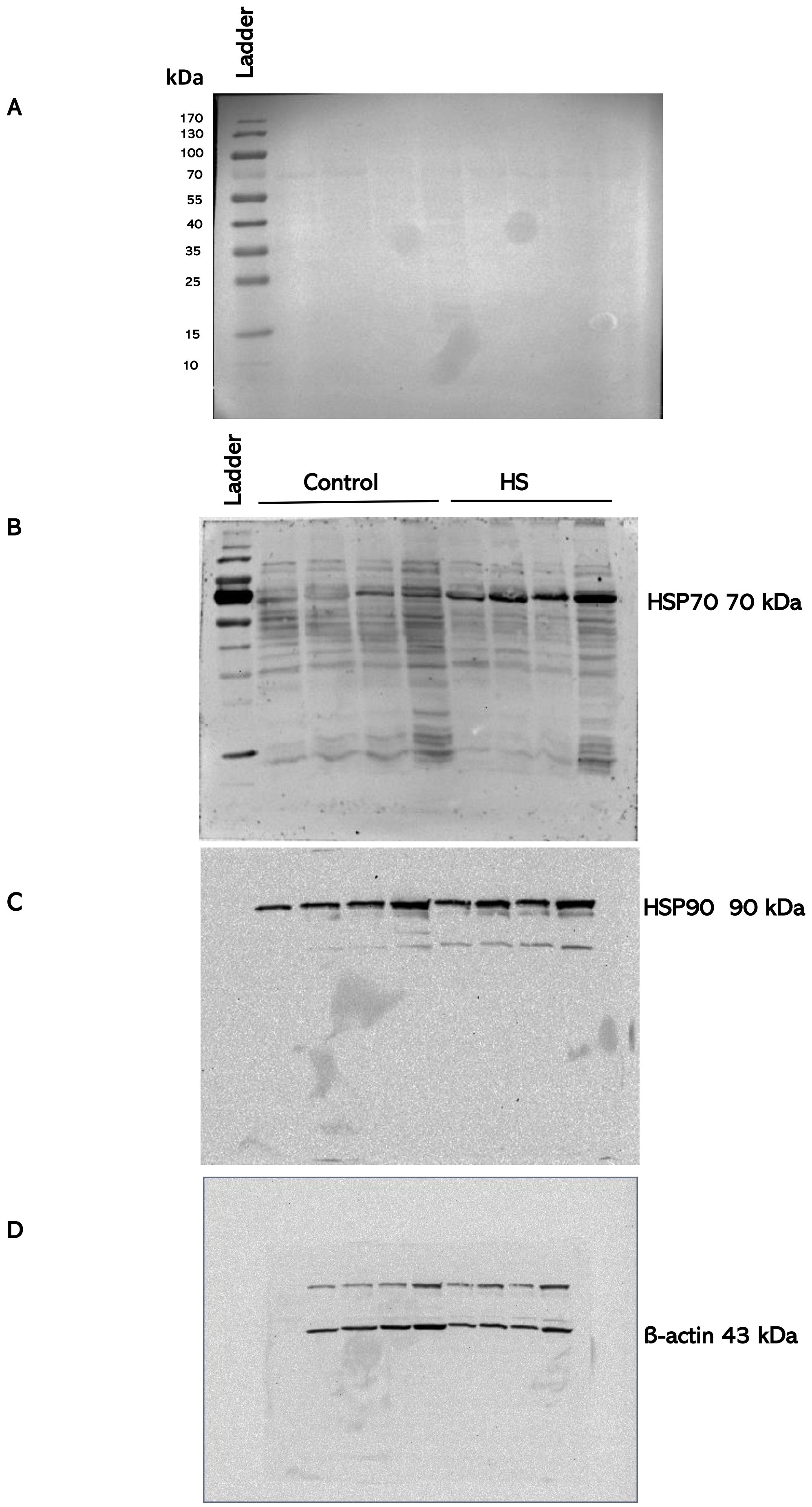
A
B
C
D
Ladder
kDa
170
130
100
70
55
40
35
25
15
10
Control
HS
HSP70 70 kDa
HSP90 90 kDa
ß-actin 43 kDa

E

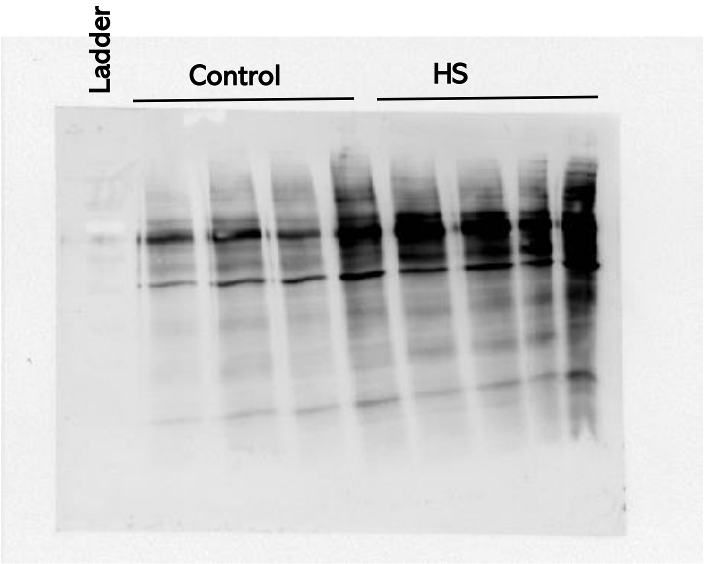

Supplementary Figure S5: Western blot analysis of HSPs and the total oxidized proteins in granulosa cells subjected to HS. (A) Molecular size ladder used with the corresponding sizes. **(B)** Full blot image of HSP70 in granulosa cells subjected to heat stress. **(C)** Full blot image of HSP90 in granulosa cells subjected to heat stress. **(D)** Full blot image of ß-Actin in granulosa cells subjected to heat stress. **(E)** Full bot image of the total oxidized proteins in granulosa cells subjected to heat stress. The 4 left lanes adjacent to the ladder represent the quadruplicates of the control group and the last 4 right lanes represent the quadruplicates of the HS group.

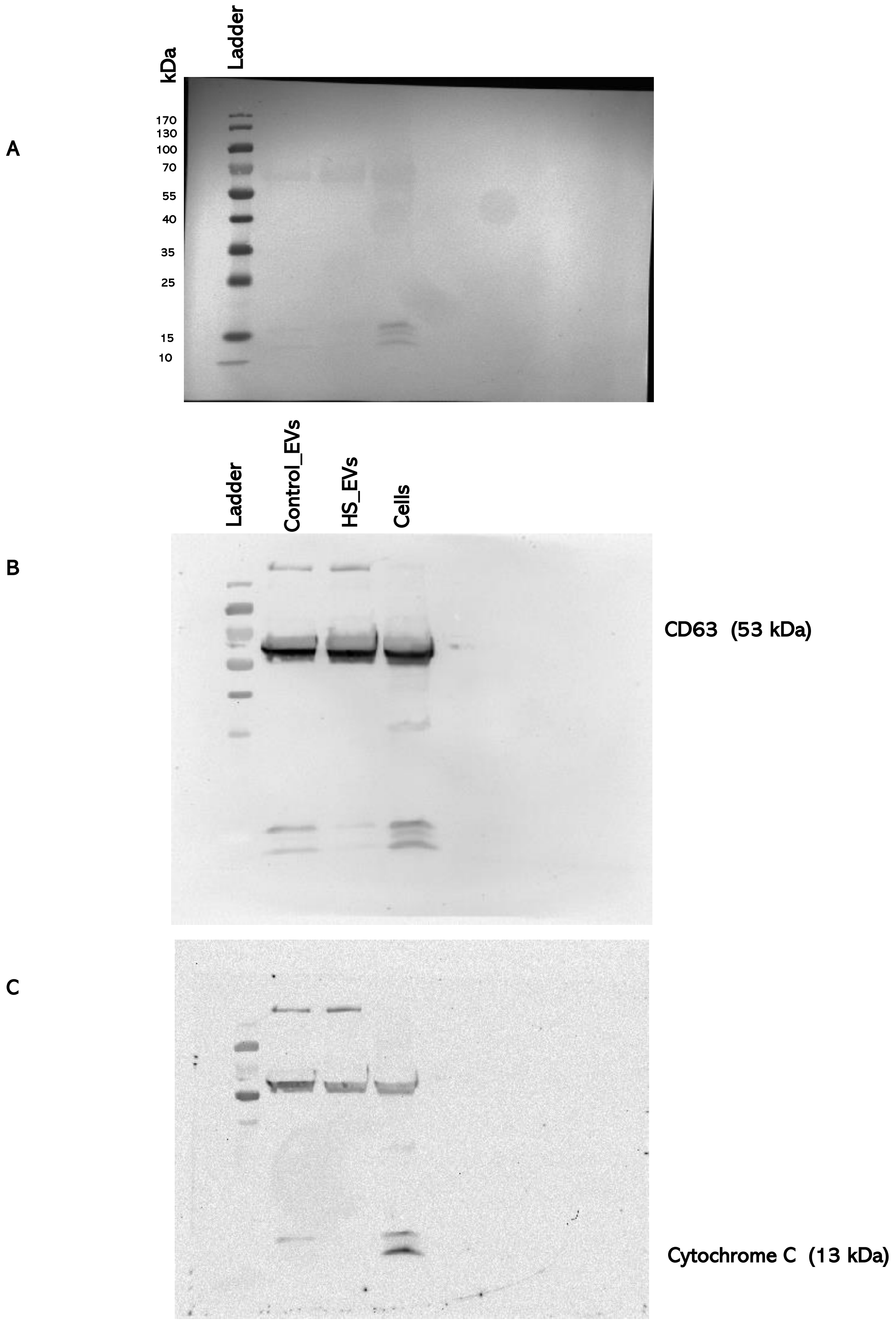
A
kDa
Ladder
170
130
100
70
55
40
35
25
15
10
B
Ladder
Control_EVs
HS_EVs
Cells
CD63 (53 kDa)
C
Cytochrome C (13 kDa)

D

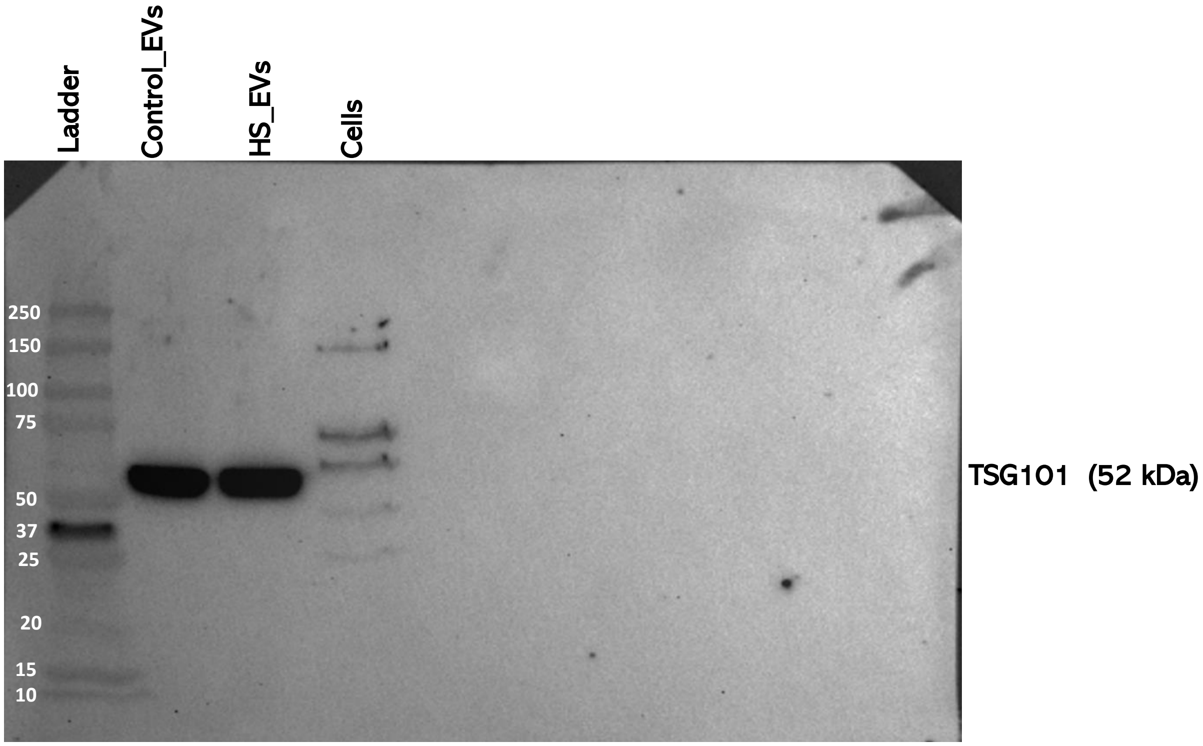
Ladder
Control_EVs
HS_EVs
Cells
250
150
100
75
50
37
25
20
15
10
TSG101 (52 kDa)

E

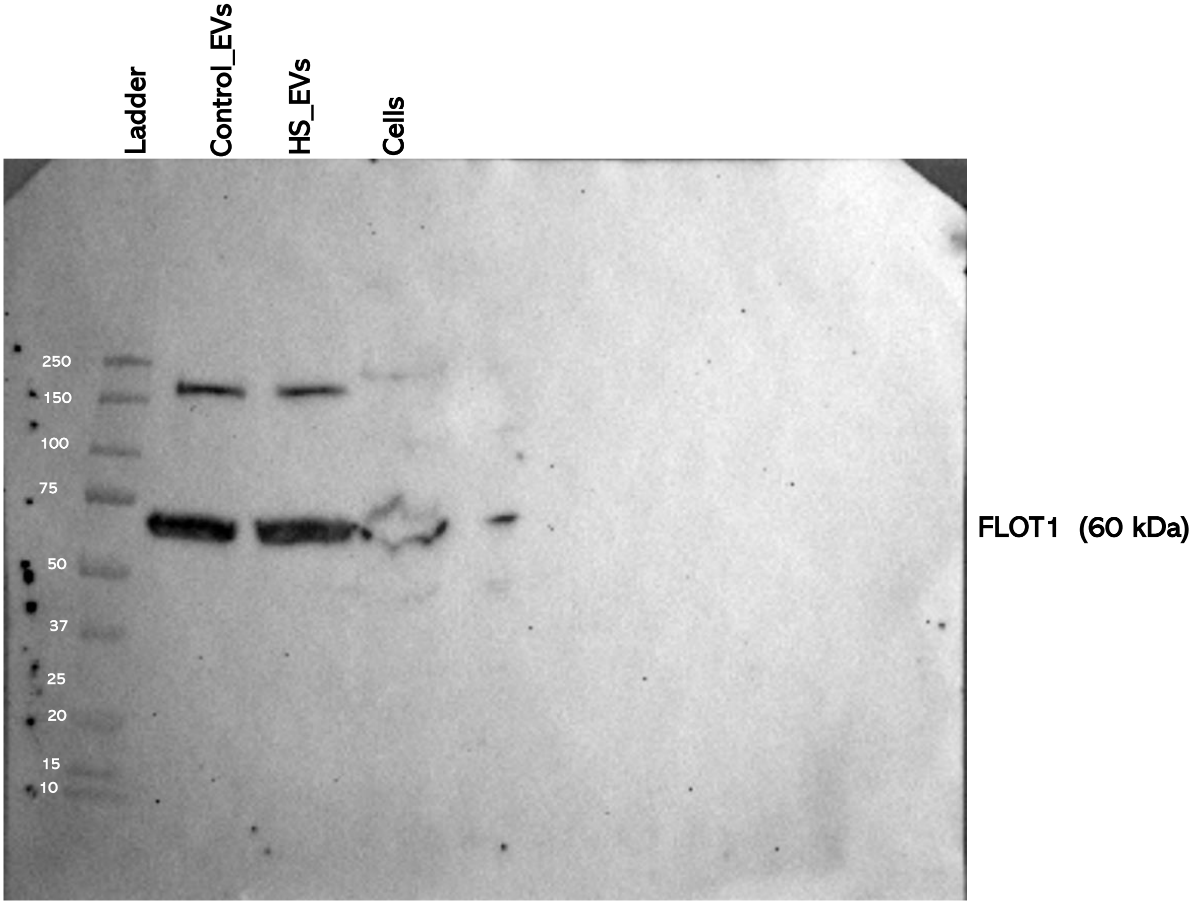
Ladder
Control_EVs
HS_EVs
Cells
250
150
100
75
50
37
25
20
15
10
FLOT1 (60 kDa)

Supplementary Figure S6: Western blot analysis of EVs positive and negative marker proteins. (A) Molecular size ladder used with the corresponding sizes. **(B)** Full blot image of CD63 protein in EVs and cell lysate. **(C)** Full blot image of Cytochrome C protein in EVs and cell lysate. **(D)** Full blot image of TSG101protein in EVs and cell lysate. **(E)** Full blot image of FLOT1 protein in EVs and cell lysate. The left lane adjacent to the ladder represents the Control_EVs followed by HS_EVs and cell lysate.
